# Fear learning induces synaptic potentiation between engram neurons in the rat lateral amygdala

**DOI:** 10.1038/s41593-024-01676-6

**Published:** 2024-06-13

**Authors:** Marios Abatis, Rodrigo Perin, Ruifang Niu, Erwin van den Burg, Chloe Hegoburu, Ryang Kim, Michiko Okamura, Haruhiko Bito, Henry Markram, Ron Stoop

**Affiliations:** 1 Department of Psychiatry, Center for Psychiatric Neuroscience, University Hospital of Lausanne, Prilly-Lausanne, Switzerland; 2 Brain-Mind Institute, Ecole Polytechnique Fédérale de Lausanne, Lausanne, Switzerland; 3 Department of Neurochemistry, The University of Tokyo Graduate School of Medicine, Tokyo, Japan

**Keywords:** Neural circuits, Fear conditioning, Amygdala

## Abstract

The lateral amygdala (LA) encodes fear memories by potentiating sensory inputs associated with threats and, in the process, recruits 10–30% of its neurons per fear memory engram. However, how the local network within the LA processes this information and whether it also plays a role in storing it are still largely unknown. Here, using ex vivo 12-patch-clamp and in vivo 32-electrode electrophysiological recordings in the LA of fear-conditioned rats, in combination with activity-dependent fluorescent and optogenetic tagging and recall, we identified a sparsely connected network between principal LA neurons that is organized in clusters. Fear conditioning specifically causes potentiation of synaptic connections between learning-recruited neurons. These findings of synaptic plasticity in an autoassociative excitatory network of the LA may suggest a basic principle through which a small number of pyramidal neurons could encode a large number of memories.

## Abstract (web summary)

A sparsely connected network, organized in clusters, identified in the rat lateral amygdala shows potentiation between recruited neurons after fear conditioning. This implies a mechanism for encoding multiple memories with a small number of neurons.

## Main

Since the seminal publications of LeDoux and Shinnick-Gallagher^1,2^, the potentiation of converging sensory inputs into the lateral amygdala (LA) has become the principal working model for how synaptic plasticity in sensory afferents underlies fear learning^3^. More recently, it was shown how fear learning recruits a subset of 10–30% of LA neurons into the fear memory engram by competitive selection based on their intrinsic excitability^4–6^. Hence, this has become a framework for studying how memories could be locally encoded within the LA^7^. However, little is known about the local connections between these neurons and whether and how they are affected by fear learning.

Recently, neuronal ensembles, that is, groups of neurons with simultaneous activity emerging from local excitatory connections, have started to receive increasing attention for their possible role in information processing and memory encoding (for a summary, see refs. ^8,9^). Thus, Jonas’ group characterized in vitro excitatory connections in the hippocampus CA3 (ref. ^10^) as well as their capability for local plasticity^11^. Further, in the developing neocortex, neurons with similar developmental origin appear to exhibit higher connectivity^12^, and in the mature visual cortex, cortical neurons with similar stimulus feature selectivity are clonally related^13^ and show higher interconnectivity^14^. However, it is not known whether this higher interconnectivity is induced by learning^12–14^. Indeed, none of the present studies seem to have directly tested the hypothesis that learning itself can induce synaptic changes in local connections within neuronal ensembles^9^. This gap in knowledge may be inherent to the limitations of the currently available chemo/opto/fluorescence viral tools that either require different neuronal genotypes or sufficient distance between pre- and postsynaptic elements to spatially tag these separately. Also, optical imaging methods provide limited temporal resolution.

In the present study, we used 12-patch-clamp single-cell electrophysiological recordings in vitro and ex vivo and 32 fine wire electrodes mounted on microdrives for single-unit recordings in vivo, together with activity-dependent expression of fluorescent and optogenetic markers to selectively study changes in synaptic connections between fear memory-recruited neurons. We found that, (1) in vitro, Hebbian stimulation can induce synaptic plasticity between local LA neurons, (2) ex vivo, after fear learning, connections between fluorescently labeled neurons are increased in synaptic strength, and, (3) in vivo, fear learning induces increases in functional connectivity between optogenetically labeled neurons. These changes in synaptic connectivity that occur specifically between engram neurons in the LA suggest that encoding of fear memories may also take place by changes within local excitatory circuitry of the LA.

## Results

### Sparse connectivity within the LA

**Fig. 1:**
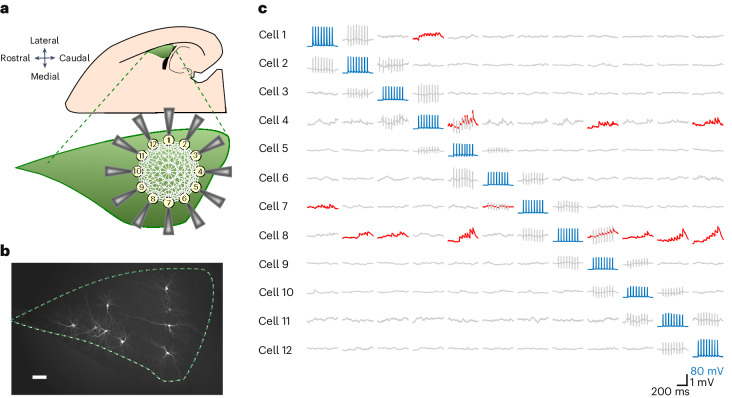
Schematic view of 12 whole-cell patch-clamp recordings in acute horizontal brain slices and post hoc fluorescent biocytin staining. **a**, The preparation involved half-hemisphere horizontal slices from a 2- to 3-week-old rat that contained the LA (green), which is easily recognizable by being bordered by the external capsule on the lateral border and the hippocampus and the lateral ventricle on the caudal border. After gaining whole-cell access to up to 12 neurons at a time, electrophysiological recordings were performed. **b**, Post hoc staining of ten biocytin-labeled neurons in the LA (*n* = 90 slices). Scale bar, 100 μm. **c**, A 20-Hz train of eight APs, in addition to a follow-up single AP (not shown), were elicited successively in each recorded neuron (blue traces) while recording spontaneous activity from the remaining neurons. Time-locked evoked responses (red traces) indicated a direct synaptic connection. Columns showing sequentially evoked APs (in blue) in cells 1–12 by injections of a 2 nA 3 ms^–1^ current and simultaneous membrane potentials (in gray) of nonstimulated cells. uEPSPs (in red) were averaged over 15 trials recorded in 14- to 19-day-old Wistar rats of both sexes.

**Fig. 2:**
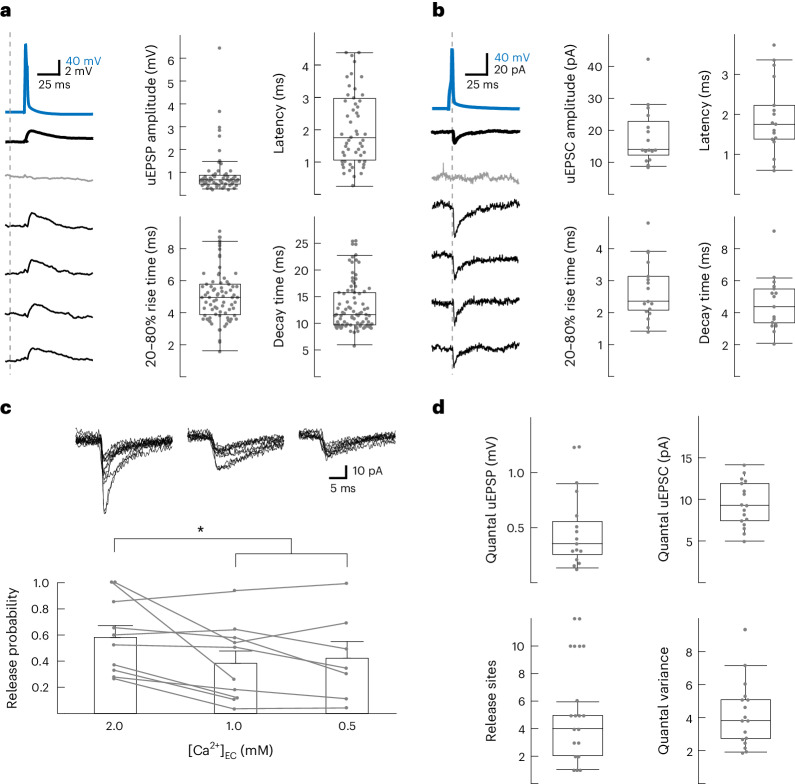
Functional connectivity within the LA. **a**, Left: Example of a presynaptic AP (blue) evoking a uEPSP (bold = 15-trial average, including failures) with individual traces (one failure in gray and four successes). Right: uEPSP characteristics (*n* = 81 connections), excluding failures. Centers represent medians, and whiskers represent 1.5× interquartile range. **b**, As in **a** but with unitary evoked postsynaptic current (uEPSC). Centers represent medians, and whiskers represent 1.5× interquartile range. **c**, Release probability as a function of extracellular Ca^2+^ concentration. Top: Ten example traces per connection. Data were analyzed by repeated measures analysis of variance (RM-ANOVA), *F*_2,15_ = 5.411, *P* = 0.017 and Tukey corrected *P = 0.033 or *P = 0.0349 for 2 mM versus 1 mM and 0.5 mM, respectively. Bar graphs show mean + s.e.m.; [Ca^2+^]_EC_, extracellular Ca^2+^ concentration **d**, Quantal parameters extracted based on a simple binomial model (Methods). Centers represent medians, and whiskers represent 1.5× interquartile range; *N* = 85 rats; Recordings were acquired in 14- to 19-day-old Wistar rats of both sexes.

**Extended Data Fig. 1:**
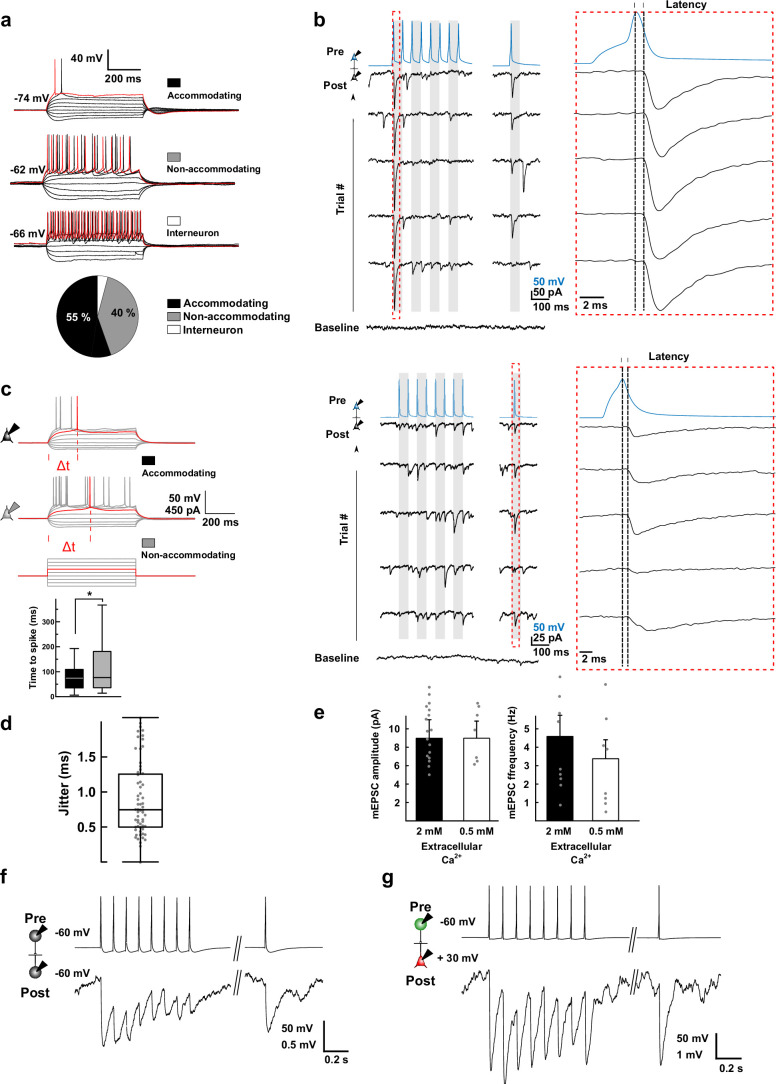
Electrophysiological characterization of neurons and connections. **a**, Examples of neuronal response patterns to 400 ms square-pulse current injection steps (50 pA), with pie chart for distribution of recorded neuron types (n = 637 neurons). Red trace represents the first current injection that triggered an action potential. **b**, Example of voltage-clamp recording with a confirmed connection between presynaptic (blue) and postsynaptic (black) neuron that was depressing (top) or facilitating (bottom); APs were elicited presynaptically (blue) and unitary excitatory postsynaptic currents (EPSCs) recorded postsynaptically (black; 5 example traces are shown); red box inset: time-scale expansion showing that uEPSC selection was time-locked to presynaptic AP peak, with a fixed latency between 0.5 to 4.5 ms. **c**, The time to spike was used as a measure of the degree of accommodation for recorded neurons, measured as the delay (Δt) to observe the first action potential upon application of minimal current injection (400 ms square pulse); Time to spike of shorter duration for accommodating neurons (top example trace) when compared to non-accommodating neurons (bottom example trace). Bar graph, Time to spike was shorter for accommodating when compared to non-accommodating neurons; Student’s t-test with Welch correction. t = -2.0386, df = 53.205, *P* = 0.04647 (*), n = 40 vs 59 neurons. Box plot indicates mean (central line), 25 and 75% interquartile, and maximum and minimum values (whiskers). **d**, Jitter for monosynaptic connections (n = 81). Box plot mean represents the median and whiskers represent the inter-quartile range x 1.5. **e**, External Ca^2+^ concentration does not influence mEPSC amplitude, whereas EPSC frequency tends to decrease with lower [Ca^2+^], indicating presynaptic modulation of vesicle release. n = 17 and 8 connections for 2 mM and 0.5 mM extacellular Ca^2+^, respectively. **f**, Example trace of one of 21 inhibitory neurons that were found in naïve slices (from rats not subjected to fear conditioning) exhibiting high spiking frequencies (>30 Hz) and high cell membrane resistances (1140 ± 114 MΩ). 12 of these made local connections, all of which were inhibitory, as shown as amplitude IPSPs, thus confirming the validity of these electrophysiological criteria to identify local interneurons. Current clamp, with baseline at -60 mV for both pre- and postsynaptic neurons. Average of 15 traces. **g**, Example trace of one out of 18 inhibitory neurons found among CFC-recruited *Arc*^+^ (GFP^+^) neurons, (same criteria as for f), 4 of these made local connections with non-recruited pyramidal neurons; all of these were again inhibitory. Current clamp, with baseline at -60 mV for the presynaptic neuron and +30 mV for the postsynaptic neuron (to increase observed inhibitory postsynaptic potential amplitude). Average of 15 traces.

To expose and characterize excitatory synapses between putative principal LA neurons in vitro, we probed with 12-pipette whole-cell patch-clamp recordings in rat horizontal brain slices for unitary excitatory postsynaptic potentials (uEPSPs) evoked by presynaptically induced action potentials in a single connection (APs; Fig. 1). This configuration allowed us to identify, per experiment, up to 132 neuronal pairs (*n*(*n* – 1)) and revealed, between 637 recorded excitatory neurons, 89 excitatory connections distributed evenly across left and right hemispheres (*n* = 47 and 43 slices, respectively; *N* = 34 rats; Fig. 2a,b, Extended Data Fig. 1a–d and Supplementary Note 1). The calculated 2.1% connectivity level (89/4,157 tested connections; Methods) places the LA between rat hippocampal CA3 (0.9%), the piriform cortex (1.0%) and primary sensory cortices (>10%)^10,15,16^. Quantal size and number of release sites (460 ± 320 µV; 5 ± 3 (mean ± s.d.); *n* = 15 trials × 17 connections; Fig. 2c,d and Extended Data Fig. 1e) were similar to CA3 (500 ± 300 µV; 3.2 ± 0.8)^10^ and the primary sensory cortex (211 ± 65 µV; 3.4 ± 2.2)^17^.

**Fig. 3:**
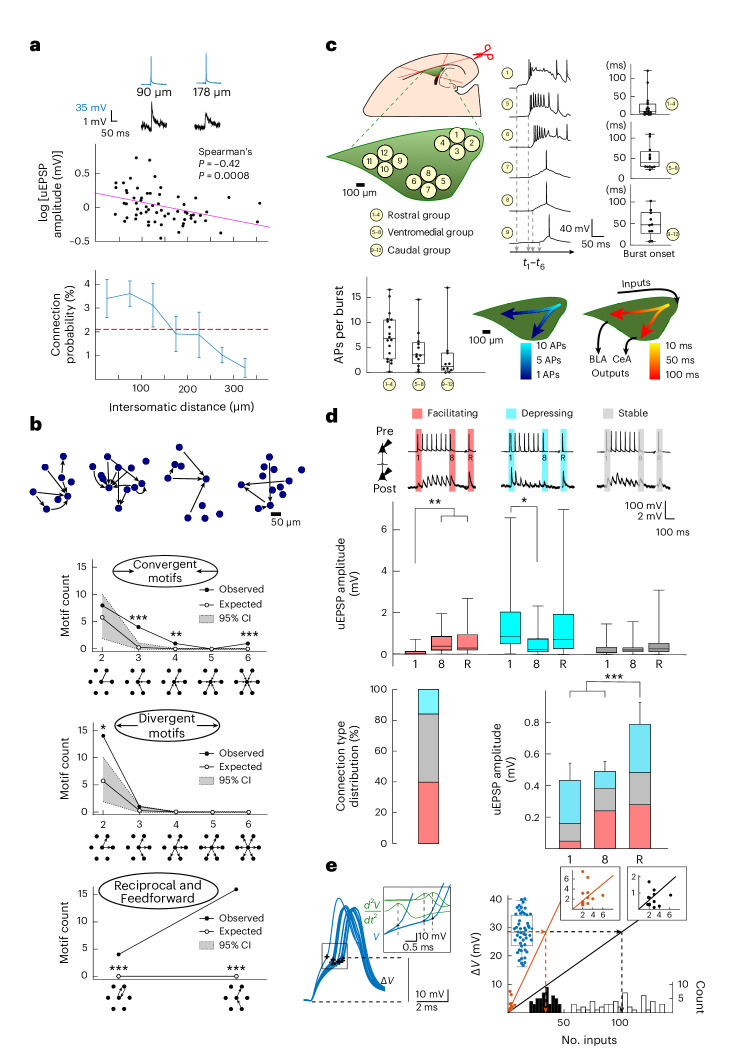
LA network organization and intra-LA signal propagation. **a**, uEPSP amplitude and connection probability decrease over distance. Inset: Examples of AP–uEPSP pairs (637 neurons, 89 connections, 34 rats; *P* = 0.0008). **b**, Top: Diverse examples of motifs with preserved cell positions. Bottom: observed (black circles) and expected (white circles) connectivity motifs (100,000 Monte Carlo simulations; Methods and Supplementary Note 3; gray, 95% confidence interval (95% CI)); *, **, and *** represent outside the 95%, 99% and 99.9% confidence intervals around the simulated values (line connecting the white dots), respectively. **c**, Top: Recordings in three LA (green) regions, with example bursting activity and corresponding averaged burst onsets per group of pipettes (*n* = 6 experiments with 18, 12 and 10 connections in clusters 1–4, 5–8 and 9–12, respectively). Bottom left: APs per burst per region. Bottom right: overlays of APs per burst and burst onset (Supplementary Note 4). Box plots indicate mean (middle line), 25% and 75% quartiles and maximal and minimal values (whiskers). BLA, basolateral amygdala; CeA, central amygdala. *t*_1_–*t*_6_, times of burst onsets for the samples. **d**, Top: Examples of facilitating, depressing and stable connections (*n* = 29, 12 and 41, respectively) and uEPSP amplitudes (average of 15 traces including failures; data were analyzed by RM-ANOVA; facilitating: *F*_2,56_ = 24, **P = 0.0045 (stimulus 1 versus stimulus 8 or 0.0015 (stimulus 1 versus stimulus 9) after Bonferroni correction); depressing: *F*_2,22_ = 7, *P = 0.032 Bonferroni corrected; stable: *F*_2,93_ = 2; *P* > 0.05). Box plots indicate mean (middle line), 25% and 75% quartiles and maximal and minimal values (whiskers). Bottom left: Connection type distribution. Bottom right: Average uEPSP depolarization per input, with relative contribution of facilitating phenotypes (RM-ANOVA; *F*_2,144_ = 11, *P* < 0.0001; ***P = 0.0002 and ***P = 0.009 for stimulus 9 versus stimulus 1 and versus stimulus 8, respectively, after Bonferroni correction; *n* = summed connections for 1, 2–8 and recovery (R) uEPSP from the top). Bars show mean ± s.e.m. **e**, Left: Voltage threshold analysis for AP initiation (ramp protocol; black, AP threshold). Inset: Calculation of time of AP initiation based on apex of second derivative. Right: Evoked uEPSP amplitudes against the number of inputs of convergent motifs (black circles, uEPSP1; red circles, uEPSPR). Insets: Magnification of the origin. A linear regression line was plotted from the summed convergent motif uEPSPs. Mean AP threshold values (blue circles) are projected (dashed line) on either regression line to construct the histogram distribution of inputs required to trigger a postsynaptic AP with either uEPSP1 (black) or uEPSPR (red); see Supplementary Note 5. Data are from the connections presented in **d**. Upper and lower limits of the boxes represent 75% and 25% values, with the whiskers extending to 100% and 0%. The middle lines represent the medians. Recordings were made in 14- to 19-day-old Wistar rats of both sexes.

**Extended Data Fig. 2:**
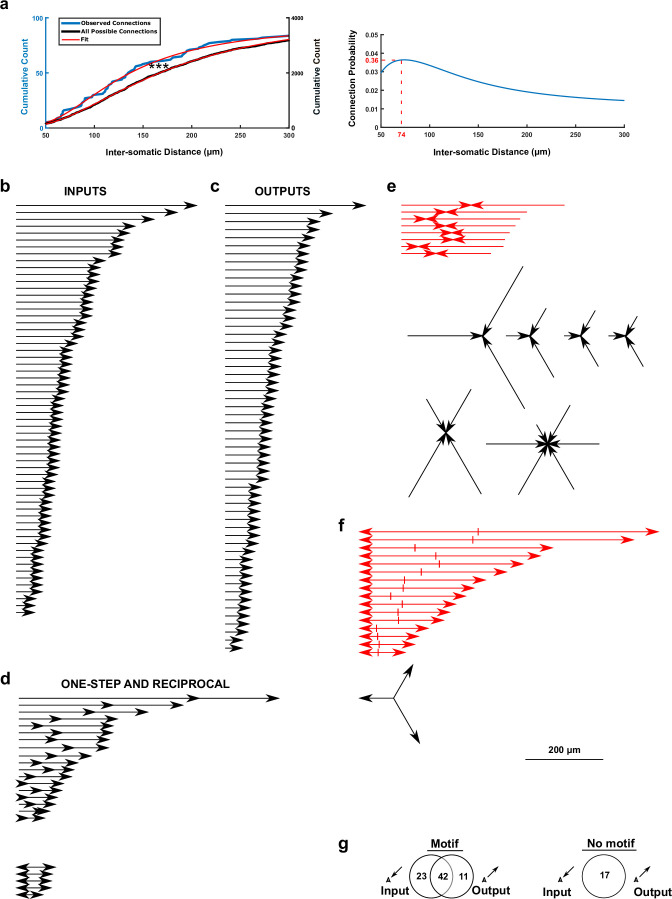
Inventory of connection distances categorized by motif. **a**, Left: Cumulative count of inter-somatic distances of observed (blue) and all possible connections (black). The respective fits (red) indicate a statistically significant left-shift for the inter-somatic distances of the observed connections (Kolmogorov-Smirnov test, D = 0.42, *P* < 0.0001 (***)). Right: The ratio of the derivatives yielded the connection probability as a function of inter-somatic distance, revealing a high connectivity (>5%) for cell bodies within 100 µm. **b-f**, List of all observed connection distances (connection distance includes arrow tip) for **b**, single inputs, **c**, single outputs, **d**, one-step feed-forward (top) and reciprocal (bottom) motifs, **e**, convergent input motifs and **f**, divergent outputs motifs. For the red-marked motifs, the summed distance of double-convergent motifs (295 ± 20 μm) is similar to the summed distance of double-divergent motifs (335 ± 48 µm; Two-sided Welch two-sample t-test, t_19.4_ = 0.8, *P* = 0.4493; ± SD), but the variance of the summed distance is an order of magnitude lower for double-convergent motifs, at 3241 μm^2^, compared to that of double-divergent motifs, at 37145 μm^2^ (Bartlett test of homogeneity of variances, K2 = 9, *P* = 0.027). This suggests that convergent inputs define a local-cluster limit (within a ~ 300 µm radius) for local processing, whereas divergent outputs can be found both locally and distantly for processing of both intra- and inter-cluster information. Scale bar applies to b-f. **g**, Venn diagrams depicting single inputs and single outputs that were part (left) or not part (right) of non-convergent respectively non-divergent motifs. In total, more than >75% (=(23 + 42 + 11) / (23 + 42 + 11 + 17)) connections were part of a motif.

By further analysis of intersomatic distances, we uncovered that the highest connection probability and synaptic strength occurred within a 100-µm radius. Both rapidly declined with increasing distance (Fig. 3a and Extended Data Fig. 2a), as also reported in rat somatosensory cortices^16^, although not in the CA3 (ref. ^10^) and piriform cortex^15^. From this distribution, we computed (Supplementary Note 2) an average of up to 230 local inputs per neuron. We found connections occurring for >75% in complex motifs, particularly and significantly more in double-divergent (14 × 2/89), triple-convergent (4 × 3/89), reciprocal (4/89) and feed-forward (16/89) motifs than expected from a random connectivity model (Fig. 3b, Extended Data Fig. 2b–g and Supplementary Note 3). Double-convergent motifs remained within a 300-µm radius around the receiving neuron, and divergent motifs extended further out (Extended Data Fig. 2e,f). Together, this suggests an organization in local clusters that are connected by longer projections^18^.

### Signal processing across the LA

**Extended Data Fig. 3:**
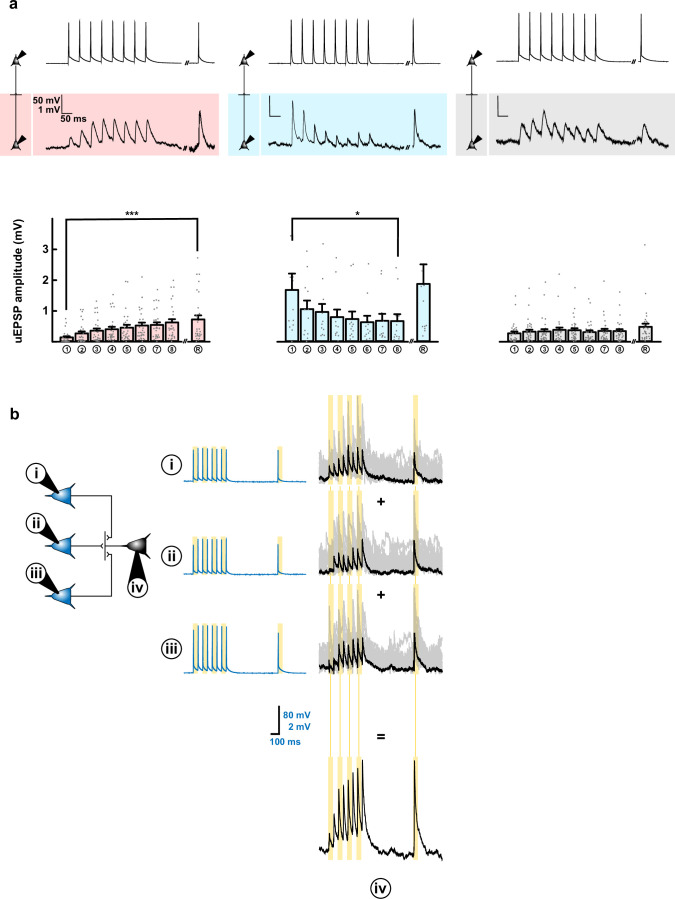
Averaged amplitudes and stability of facilitating, depressing and stable connections, and input summation at LA-LA synapses. **a**, Connections could be characterized as facilitating (red), depressing (blue) or stable (grey). Top, examples for each connection type, with each example displaying the respective recordings of presynaptic action potentials and postsynaptic uEPSPs (average of 15 sweeps). Bottom, Averaged uEPSP amplitudes for 1st through 8th and ‘recovery’ stimuli; for n = 29 facilitating, 12 depressing and 41 stable connections providing additional information to Fig. 3d. Friedman test on repeated measures with Bonferroni post-hoc comparisons. * *P* = 0.019, *** *P* < 0.0001. Bars are means + SEM. **b**, Example of input summation at a triple convergent motif. Three presynaptic neurons (blue traces and blue cells; numbered i, ii and iii) converge onto one postsynaptic neuron (black traces and black cell; numbered iv). Individual presynaptic stimulation of each blue cell (i-iii) at 20 Hz leads to a postsynaptic facilitating response (iv). The arithmetic summation (+signs) of all black responses leads to (=sign) the summed EPSP response that results from stimulating all connections simultaneously – see^43^. This convergent motif is an example of the summed EPSPs that are used in Fig. 3e (right panel; black and red dots).

We further characterized the functional organization of this LA network by inducing glutamatergic epileptiform bursts with bicuculline perfusion (Methods)^19,20^. The variability in onset and spreading of bursts (measured with 3 × 4 groups of patch pipettes) indicated a local buildup propagating in caudal to medial and rostral directions, that is, from the LA to its output regions in the central and basal amygdala, respectively. Furthermore, this was accompanied by a decrease in APs, suggestive of progressive filtering (Fig. 3c and Supplementary Note 4). To further study the filtering characteristics of this network, we exposed individual connections to a series of repetitive stimulations (eight presynaptic APs at 20 Hz). This revealed 33% facilitating, 46% stable, 13% depressing and 8% uncategorized types of synaptic contacts. All exhibited a full recovery uEPSP (uEPSPR) 500 ms later, leading to an overall temporal summation of 183% (*n* = 89; Fig. 3d and Extended Data Fig. 3a). Based on an arithmetic spatial summation within this network (Fig. 3e, Extended Data Fig. 3b and Supplementary Note 5), this would imply the requirement of synaptic inputs of 100 ± 23 to trigger a postsynaptic AP which, as a result of temporal summation during 20-Hz stimulation, would reduce to a total of 34 ± 8. The local LA network can thus act as a high-pass filter that propagates APs during heightened activation of a sufficient number of synapses. Based on previous findings that cued fear conditioning (CFC) recruits 10–30% of LA neurons^6^ (which corresponds to activation of 23–69 of a total of 230 neuronal inputs; see above and Supplementary Note 2) and on the premise that CFC can indeed induce a local synaptic potentiation that reaches a similar level as temporal summation, this should allow for a reliable signal propagation across the LA^21,22^.

### In vitro potentiation of connections

**Fig. 4:**
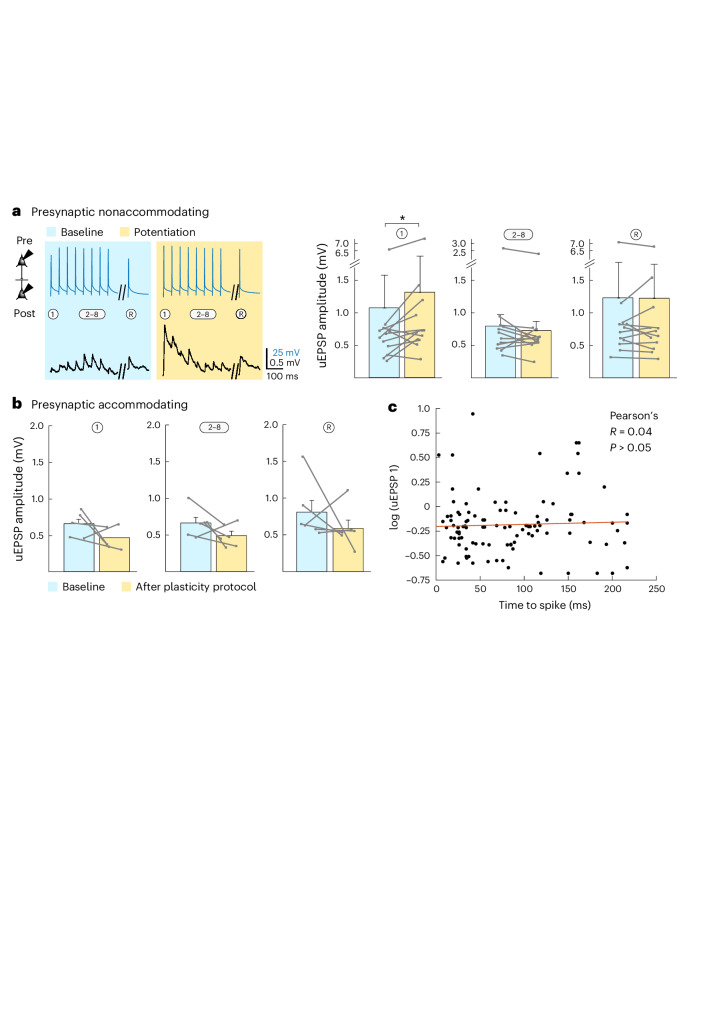
In vitro synaptic plasticity between LA neurons. **a**, In vitro-induced potentiation between connections that involve presynaptic nonaccommodating neurons before (blue) and after (yellow) the Hebbian association protocol. Left: Example trace of averaged AP-evoked (top) uEPSPs (bottom) for one connection taken 5 min before and 10 min after the induction protocol. Right: Average normalized amplitudes of 1, 2–8 and R uEPSPs excluding failures. Data were analyzed by two-tailed paired Wilcoxon signed-rank test (uEPSP 1: *W* = 10, *P = 0.042; uEPSP 2–8: *W* = 20, *P* = 0.303; uEPSP R: *W* = 32, *P* = 1; all with Bonferroni correction for multiple comparisons; *n* = 11 connections). Data are shown as mean ± s.e.m. **b**, Average normalized amplitudes of 1, 2–8 and R uEPSPs, excluding failures. Unlike connections with a presynaptic nonaccommodating neuron, connections with a presynaptic accommodating neuron did not potentiate. Data were analyzed by two-tailed, paired Wilcoxon signed-rank test (uEPSP 1: *W* = –13, *P* = 0.2188; uEPSP 2–8: *W* = –14, *P* = 0.1875; uEPSP R: *W* = –19, *P* = 0.0625; all Bonferroni corrected, *n* = 6 connections). Data are shown as mean ± s.e.m. **c**, Synaptic strength could not be predicted by presynaptic time to spike, as this did not correlate with uEPSP 1 amplitude before the induction of plasticity. This suggests that before fear conditioning, neurons that are prime candidates to be recruited into the fear memory trace do not have stronger local connectivity. Recordings were made in 14- to 19-day-old Wistar rats of both sexes.

**Extended Data Fig. 4:**
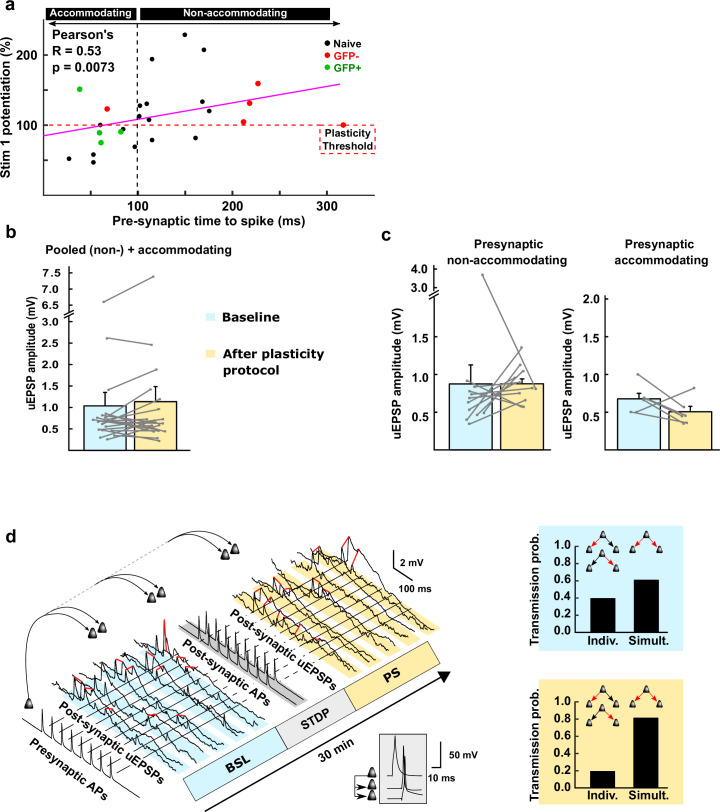
Synaptic potentiation in different types of connections. **a**, Pre-synaptic non-accommodating neurons (characterized by their long time to spike, see M&M and Extended Data Fig. 1c) were more likely to develop a potentiation of uEPSP1 (Stim1 potentiation) upon application of the Hebbian plasticity induction protocol; naïve (black) connections refer to Fig. 4a, whereas green and red connections refer to Fig. 5a–d, with green dots reflecting uEPSP1 amplitude in CFC-recruited (GFP-positive) and red dots in non-recruited neurons (GFP-negative). **b**, Pooling uEPSP1 amplitudes of neuronal pairs with either a presynaptic non-accommodating or accommodating neurons did not reveal potentiation (Two-tailed Wilcoxon signed-rank test, W = 71, *P* > 0.05, n = 20). Error bar is mean + sem. **c**, Left: there was no change in overall uEPSP amplitude across the stimulus train (uEPSP1-8 through uEPSPR, two-sided paired Student’s t-test, t_5_ = 0.8, *P* > 0.05, n = 12 connections with a pre-synaptic non-accommodating neuron), suggesting that a redistribution of synaptic efficacy – a presynaptic mechanism – explains the potentiation of uEPSP1; right: overall uEPSP amplitude after Hebbian pairing for connections with a pre-synaptic accommodating neuron (uEPSP1-8 through uEPSPR, paired Student’s t-test, t_5_ = 1.7, *P* > 0.05, n = 6). **d**, A double-output motif subjected to the Hebbian protocol for inducing synaptic plasticity. Left, uEPSPs recorded from the double-output motif, before (blue) and after (yellow) Hebbian pre- and post-synaptic pairing (grey, zoomed inset) of 10 pre- and post-synaptic (within 10 ms delay) APs at 30 Hz, repeated 15 times, with an inter-trial interval of 10 s (as used in Fig. 4a); pairing; uEPSPs were considered to occur either simultaneously (‘Simult.’; both connections; red lines) or individually (‘Indiv.’; exactly one connection, but not both) between the two connections of the motif. Right, the normalized probability for simultaneous EPSPs was higher after Hebbian pairing, when compared to individual release, indicating synchronization of neuronal activity.

**Extended Data Fig. 5:**
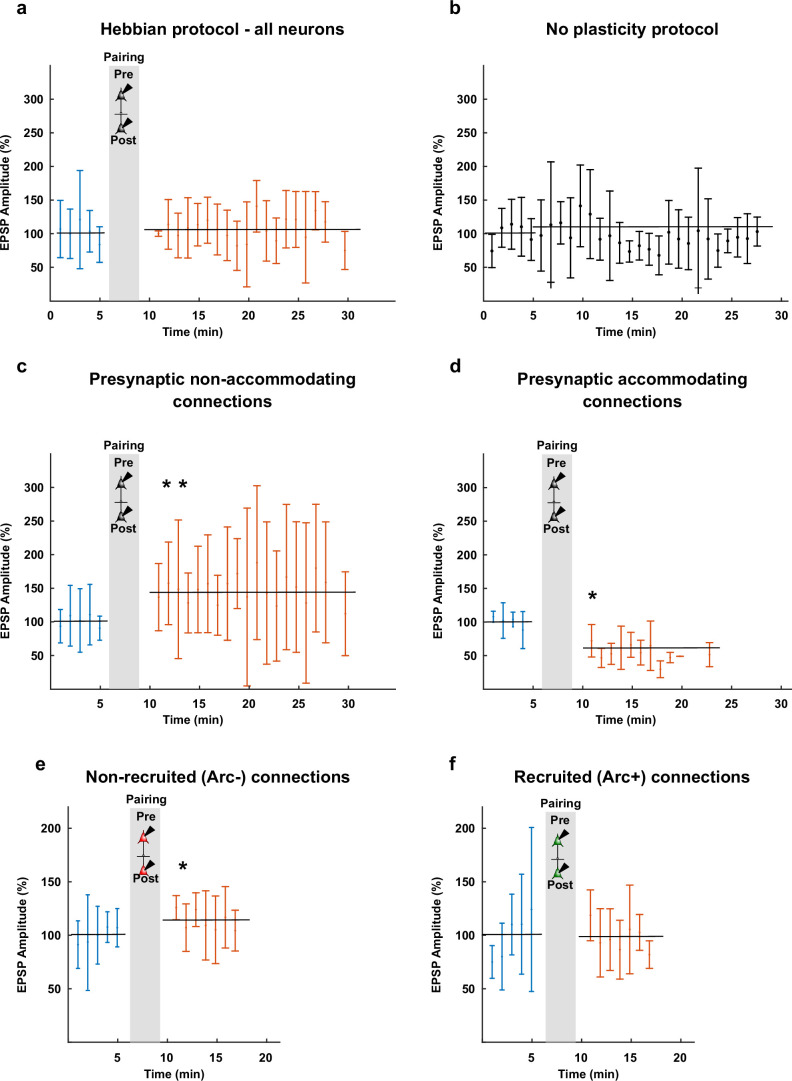
Long-term measurements of synaptic strength. **a**, Average (n = 20) long term recording of evoked EPSP amplitudes (normalized to baseline) before (blue) and after (red) induction of changes in synaptic strength through the Hebbian pre- and postsynaptic pairing (gray). No changes were observed (1 data point = 1 min average for 3 samples every 20 seconds); Two-tailed Mann-Whitney U test on the five averaged values before and after the pairing, U = 12; *P* > 0.05, n = 20 **b**, Average (n = 10) long term recordings of evoked EPSC amplitudes (normalized to first 5 minutes) show that no plasticity occurs spontaneously, without the application of a Hebbian protocol; Two-tailed Mann-Whitney U test on the five averaged values before and after the pairing, U = 7; *P* > 0.05, n = 10. **c**, as in Fig. a, including only connections with a presynaptic non-accommodating neuron; here, we observed potentiation after Hebbian pre- and postsynaptic pairing; Two-tailed Mann-Whitney U test on the five averaged values before and after the pairing, U = 0; *P* = 0.0079 (**), n = 11 **d**, as in Fig. a, including only connections with a presynaptic accommodating neuron showing depression of synaptic strength. Two-tailed Mann-Whitney U test on the four values before and five averaged values after the pairing, U = 0; *P* = 0.0159 (*), n = 6. **e**, as in Fig. a, for connections not recruited after CS-US association; Two-tailed Mann-Whitney U test on the five values before and after the pairing, U = 3; *P* = 0.0278 (*), n = 6. **f**, as in Fig. a, for connections recruited after CS-US association; One-tailed Mann-Whitney U test on the five values before and after the pairing, U = 11; *P* > 0.05, n = 4. Error bars are s.d.

To test this premise, we first assessed whether the LA network is indeed capable of local synaptic plasticity. For this purpose, we used a standard Hebbian association protocol^23^ in vitro, to associate 15 × 10 presynaptic APs (at 30 Hz) with postsynaptically evoked APs (at a delay of 10 ms). This indeed potentiated LA–LA connections by 140 ± 16% but only in intrinsically higher-excitable, nonaccommodating neurons (normalized on baseline; Fig. 4a and Extended Data Fig. 4a,b). Connections from accommodating neurons did not show any potentiation (Fig. 4b) nor did connections that were not exposed to this protocol (Extended Data Figs. 4a,b and 5a–d). The synaptic strengths of nonaccommodating and accommodating neurons were similar before exposure to the Hebbian protocol (baselines of first uEPSP from nonaccommodating (Fig. 4a) and accommodating (Fig. 4b) neurons were not significantly different; Mann–Whitney, *U* = 32, *P* = 0.7) and did not correlate with intrinsic excitability (Fig. 4c), indicating that synaptic strength does not predict potentiation. However, the excitability-based requirement for the induction of potentiation narrowly follows previous observations that intrinsic excitability favors recruitment in the fear memory engram^6^.

**Extended Data Fig. 6:**
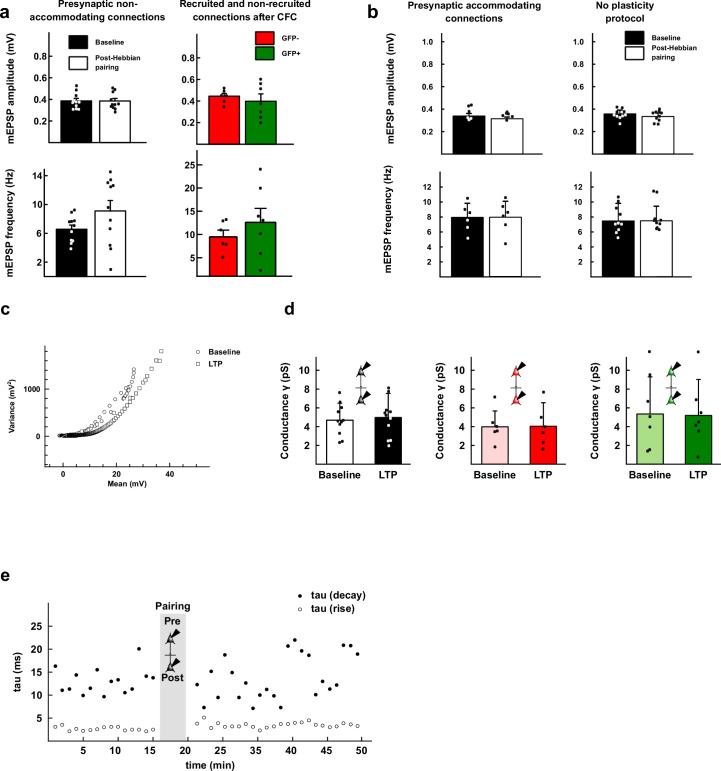
*In vitro* and *ex vivo* changes in local connection strength and probability. **a**, mEPSP frequency, nor amplitude significantly changed following Hebbian association of pre- and postsynaptic activity *in vitro* (black and white bars) when presynaptic neurons are non-accommodating nor in recruited (green) versus non-recruited (red) neurons *ex vivo*, suggesting that plasticity and CFC-mediated recruitment both have a presynaptic site of expression (Two-tailed Student’s t-tests, top-left: t_7_ = 1.2, n = 11; bottom-left: t_7_ = 1.5, n = 11; top-right: t_4_ = 0.4, n = 7 GFP^+^ and 5GFP^−^; bottom-right: t_4_ = 1.7, n = 7 GFP^+^ and 6 GFP^−^, *P* > 0.05 in all cases). Bars are means + sd. **b**, No mEPSP amplitude or frequency changes were observed in vitro when the presynaptic cell was accommodating (n = 6) or when no plasticity induction protocol was applied (n = 10). Bars are means + sd. **c**, Example relationship between voltage-variance for one connection (also known as Non-stationary fluctuation analysis): the variance of the fluctuation of the decays for each EPSP in comparison to the mean is plotted as a function of the mean EPSP decay amplitude, during baseline (circles) and after Hebbian induction (squares); **d**, The conductance (γ) is estimated from the voltage-variance relationship for each cell during baseline and after Hebbian induction of plasticity for naïve slices (left, as in Fig. 4a; n = 11), non-recruited connections (middle, red; n = 6) and recruited connections (right, green; n = 7); Two-tailed Student’s t-test, *P* > 0.05. Bars are means + sd. **e**, Rise times (20–80%, open circles) and decay times (62%, filled circles) for all EPSPs used for non-stationary fluctuation analysis. Hebbian pre- and post- synaptic pairing affected neither rise time nor decay time. To note: although we did not find any changes in mEPSP/mEPSC characteristics after changes in synaptic strength, mEPSP/mEPSC may also originate from projections outside of the LA.

Potentiation between neurons within the LA thereby provides a first basis of how local increases in connection strength could bind intrinsically higher-excitable neurons together for stronger coactivation. This potentiation seems to involve a presynaptic redistribution of synaptic efficacy toward the first stimulus^24^ without any postsynaptic changes in miniature EPSP (mEPSP) amplitude nor AMPA receptor conductance (Extended Data Figs. 4c and 6; of note, however, mEPSPs may also originate from projections outside the LA). Consistent with this redistribution of efficacy, in a double-divergent motif, we found that potentiation caused an increased co-occurrence of successful transmissions at the beginning of the train (Extended Data Fig. 4d). Taken together, this synaptic potentiation leads to a redistribution of presynaptic efficacy binding CFC-recruited LA neurons into a network that exhibits instantaneously higher response characteristics (as has also been observed in vivo after fear conditioning^21^) and a heightened sensitivity to propagate single, nonrepetitive stimuli, such as evoked by fear memory recall.

### Ex vivo connectivity after CFC

**Fig. 5:**
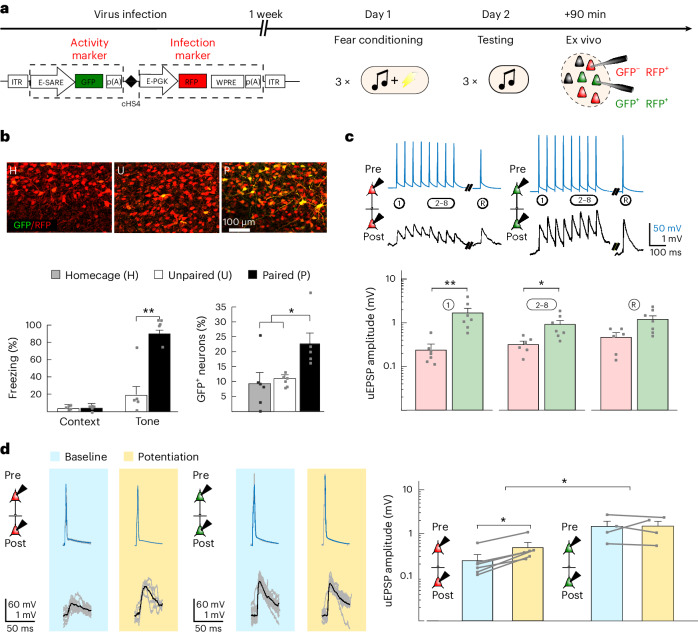
CFC (in vivo)-induced ex vivo potentiation. **a**, Virus infection protocol with stable (RFP) and dGFP (*t*_1/2_ = 2 h) under the E-SARE promoter, followed by CFC after 1 week (day 1), memory recall testing (day 2) and, within 90 min, brain extraction for imaging or electrophysiology. **b**, Top: GFP and RFP expression in LA slices of rats exposed to homecage or unpaired or paired tone–shock presentations. Bottom left: Freezing in response to the CS^+^ (one-tailed Mann–Whitney *U*-test; *U* = 0, **P = 0.0079; *n* = 5 rats). Bottom right: GFP^+^ neurons as a percentage of infected (RFP^+^) neurons (one-way ANOVA; *F*_2,14_ = 4.614, *P = 0.0075 and *P = 0.02 for paired versus unpaired and homecage after Bonferroni correction; *n* = 5 GFP^+^ neurons per slice for paired and *n* = 6 for unpaired and homecage conditions). **c**, Top: Example recordings from GFP^−^GFP^−^ (red) and GFP^+^GFP^+^ (green) connections. Bottom: Average amplitudes of uEPSPs (1, 2–8 and R) in GFP^−^GFP^−^ (red; *n* = 6) and GFP^+^GFP^+^ (green; *n* = 7) connections. Data were analyzed by two-tailed Mann–Whitney *U*-test (uEPSP1: *U* = 1, **P = 0.0051; uEPSP2–8: *U* = 3, *P = 0.017; uEPSPR: *U* = 7, *P* = 0.0653). **d**, uEPSP1 example recordings and averaged amplitudes for GFP^−^GFP^−^ (left; one-tailed paired Wilcoxon signed-rank test with Bonferroni correction; *W* = 0, *P* = 0.031, *n* = 6 connections) and GFP^+^GFP^+^ (right; *W* = 4, *P* > 0.05, *n* = 4 connections) at baseline (blue) and after the Hebbian protocol (yellow). Bar graphs show mean + s.e.m. Recordings were made in 5- to 6-week-old Sprague–Dawley rats of both sexes.

**Extended Data Fig. 7:**
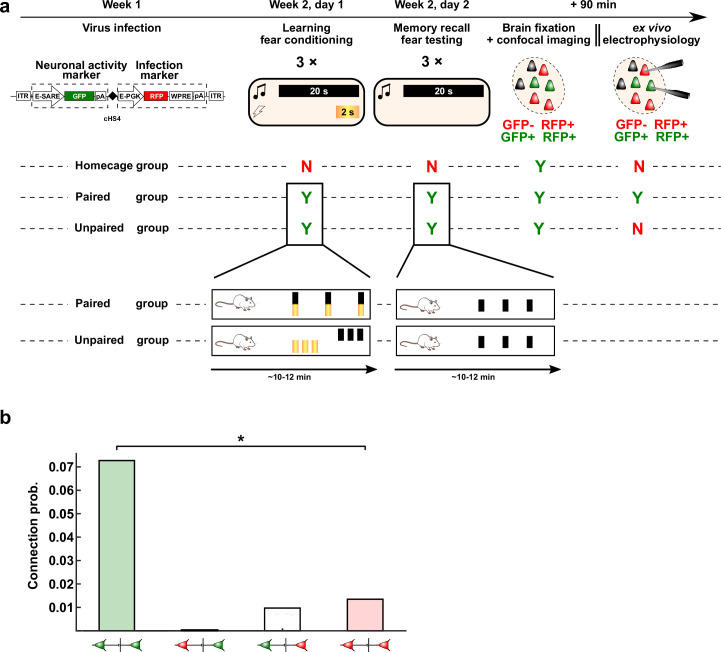
Detailed experimental outline of viral labeling of activated neurons and their connection probability following fear conditioning. **a**, Protocol timeline: from virus infusion to fear conditioning and recall of the memory (testing), followed by imaging or electrophysiology 90 min later. Viral construct: bilaterally injected with glass pipettes targeting the LA. The viral construct is flanked by Inverted Terminal Repeats (ITR), expresses d2Venus under the enhanced synaptic activity response element (E-SARE) and the red fluorescent protein (RFP; FP635) under the constitutive enhanced phosphoglycerate kinase promoter (E-PGK). The woodchuck hepatitis post-transcriptional regulatory element (WPRE) enhances expression levels and is followed by a poly-adenylation (pA) signal. Behavior: Rats in the Paired group were fear conditioned by co-terminated pairing of the conditioned stimulus (CS, 20 second tone, black bars) and unconditioned stimulus (US, electric shock, yellow bars) thrice, at random intervals (from 60 s to 180 s – determined by Matlab’s *rand* function). The Unpaired group received 3xUS and 3xCS at the start and end of the conditioning session, respectively. Fear memory recall was tested by 3xCS presentations for both Paired and Unpaired groups. The home-cage group was not exposed to CS, US or conditioning context. Rats were sacrificed 90 min. after conditioning when GFP expression is optimal. Confocal imaging: GFP^+^ and RFP^+^ neurons were counted as memory-recall-participating and/or infected, respectively. *Ex vivo* electrophysiology (Paired group): multi-electrode whole-cell patch-clamp was performed on GFP^+^ and GFP^−^ neurons to assess connectivity and connection strength of connections recruited during memory recall. **b**, Connectivity was significantly higher between (recruited) GFP^+^-GFP^+^ neurons (n = 15 slices with GFP^+^-GFP^+^ connections; 49 GFP^+^ neurons, with 6 out of 107 possible connections) than between GFP^−^-GFP^−^ neurons (n = 39 slices with GFP^−^-GFP^−^ connections; 137 GFP^−^ neurons, with 5 out of 369 possible connections) (Two-sided Wilcoxon rank sum test with continuity correction, W = 378.5, *P* = 0.0292 (*)); however, overall connectivity calculated from all connections between recruited and non-recruited neurons was unchanged at ~2%, that is similar to connectivity in naïve homecage controls (see Fig. 3a, red-dashed line), suggesting that plasticity does not increase the total number of connections within the LA.

**Extended Data Fig. 8:**
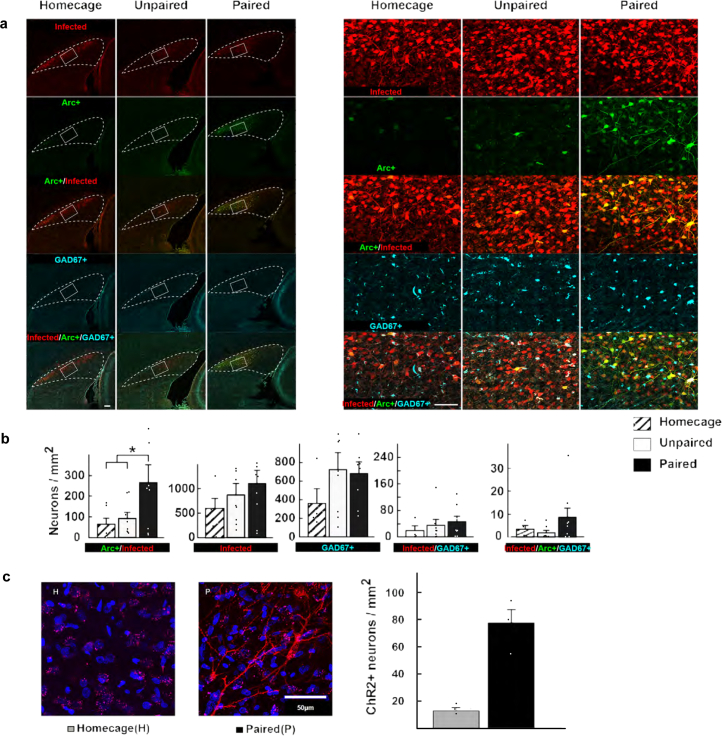
Arc expression is elevated after fear memory testing. **a**, Tiled confocal fluorescent images at 40X oil magnification of rat horizontal brain slices containing the LA (dashed white line) and showing neuronal activation across the groups exposed to homecage, unpaired fear conditioning, and paired fear conditioning. Left, distribution of infected neurons (red), infected and fear memory recall-activated neurons (Arc^+^ as revealed by GFP expression; green) and GAD67+ cells (blue). Images were obtained with maximal aperture size; white scale bar: 200 µm. Right, magnification of the LA region indicated by the white rectangle from wide-field view on the left; images obtained as maximal intensity projection of ~25 stacks of 2 µm optical slices; scale bar: 100 µm. **b**, Density of neurons expressing markers of infection, activation (Arc^+^), GABA (GAD67^+^), or combinations thereof after fear memory testing; from left to right: 1) infected and Arc^+^ neurons; 2) infected neurons; 3) GAD67^+^ neurons; 4) infected GAD67^+^ neurons; 5) infected and Arc^+^ GAD67^+^ neurons. The density of neurons expressing one of the five different combinations of markers is presented on the right as the number of neurons / mm^2^. Note that the number of Arc^+^ neurons is significantly higher in the paired group (One-way ANOVA, F_2,19_ = 3.4, *P* = 0.0289 and 0.049 after Bonferroni correction (*), n = 5 rats per group, for infected Arc+ neurons). These neurons are in large majority glutamatergic neurons, as Arc^+^ GAD67^+^ neurons are almost absent from any of the three groups (graph 5). In Fig. 5b, the number of recruited neurons (Arc^+^) was taken from this graph but normalized to the number of infected neurons to accommodate variability for infection rates across animals (N = 4 rats per group. Data are means + s.e.m.). **c**, Representative image of ChR2 expression, as revealed by fluorescence of co-expressed mCherry, and quantification of mCherry/ChR2-positive cells. The images are representatives of images from rats 1, 5, 8 and 10 that are shown in extended data 9e.

To test whether these in vitro-induced increases in strength of local connectivity in the LA could also be found ex vivo, after fear learning, we used an adeno-associated virus (AAV) expressing a destabilized form of green fluorescent protein (dGFP; *t*_1/2_ = 2 h) under the *Arc* promoter enhanced synaptic activity responsive element (E-SARE)^25^ as a reporter of recruited LA neurons after CFC (Fig. 5a and Extended Data Fig. 7a). Fear recall the next day was indeed able to efficiently induce dGFP expression 90 min later in 24% of the total neuronal LA population (Fig. 5b and Extended Data Fig. 8a,b), similar to well-known reported percentages in mice^4^ and reflecting successful reactivation of neurons using the E-SARE promoter. Subsequent ex vivo 12-patch-clamp recordings of the recruited (GFP^+^red fluorescent protein^+^ (GFP^+^RPF^+^)) LA neurons showed (compared to nonrecruited, only RFP^+^ neurons) more connections (7.2% between recruited neurons and 1.4% between nonrecruited neurons; Extended Data Fig. 7b) with higher uEPSP responses (1.67 ± 0.37 versus 0.24 ± 0.08 mV; Fig. 5c). Again, as in our in vitro findings, no changes occurred in postsynaptic efficacy (Extended Data Fig. 6a,d). Taken together, these recordings show that, after fear learning, recruited neurons in the LA are more likely to be tied together and exhibit stronger connections than nonrecruited neurons.

Hypothetically, it is possible that the increased synaptic strength that we found ex vivo between recruited neurons is, in fact, not the result of fear learning but rather reflects selective recruitment of neurons into the fear memory engram that already had stronger synaptic connections before fear learning, that is, that stronger connections between neurons serve as a selection criterion for inclusion in the fear memory engram^4,26,27^. However, this reasoning may not apply because we found no correlation between synaptic strength and excitability in naive slices, demonstrating that there is no stronger local synaptic connectivity before recruitment between neurons that exhibit higher intrinsic excitability (that is, are more likely to become potentiated in our in vitro induction protocol; Fig. 4c). Thus, it is most likely that the stronger connections between recruited neurons must have resulted from potentiation induced by CFC.

To discriminate further between these two possibilities, in brain slices prepared after the CFC protocol, we also experimentally tested whether the in vitro Hebbian-induced synaptic plasticity was occluded in recruited connections. Therefore, we compared the ex vivo CFC-induced potentiation with Hebbian-induced in vitro potentiation by applying the Hebbian in vitro protocol (compare Fig. 4a–c) on CFC-recruited and nonrecruited neurons. Whereas further potentiation was indeed occluded in the recruited neurons (uEPSP1: 1.44 ± 0.39 → 1.47 ± 0.36 mV), it was still possible between nonrecruited neurons (0.24 ± 0.08 mV → 0.47 ± 0.13 mV; Fig. 5d and Extended Data Fig. 5e,f). This suggests that, through a Hebbian mechanism, CFC leads to synaptic strengthening between recruited LA neurons and may thereby ensure, after fear conditioning, a strengthening of local synaptic connections that can promote reliable signal propagation across the LA (compare Fig. 3d,e).

### CFC-driven potentiation of connections

**Fig. 6:**
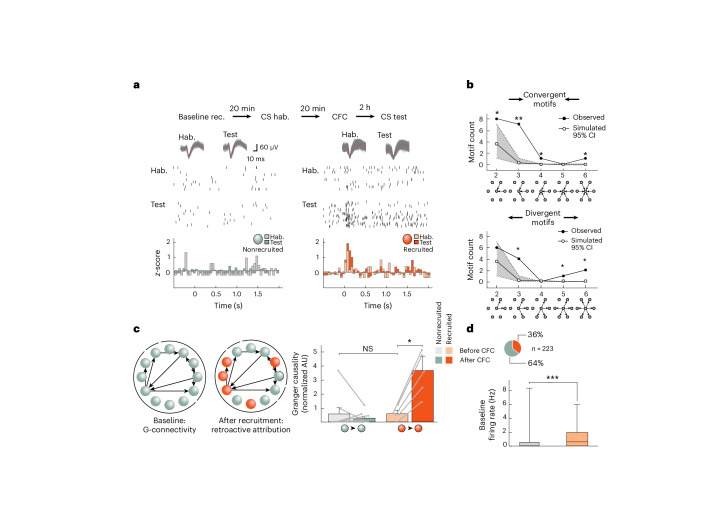
In vivo connections and plasticity between LA neurons. **a**, In vivo recordings (rec.) of neuronal spiking before (light colors) and 6 h after (dark colors) CFC (*n* = 7 rats). Top: Examples of neuronal spiking and CS^+^-evoked *z*-scores (after CFC) of recruited (orange) and nonrecruited neurons (gray); hab., habituation. **b**, Distributions of simulated (white, Monte Carlo; see Methods and Supplementary Note 3) and observed (black) connectivity patterns. The gray area around the central line indicating simulated values represents the 95% confidence interval. Single asterisks and double asterisks indicate observed values outside the 95% and 99% confidence intervals, respectively. **c**, Left: Connectivity diagrams before and after CFC. Right: Granger causality strength (normalized) for nonrecruited (*n* = 8) and recruited (*n* = 5) connections. Data were analyzed by one-tailed paired Wilcoxon signed-rank tests (nonrecruited: *W* = –3, *P* > 0.05; recruited: *W* = 15, *P < 0.05 and *P* = 0.06 (not significant (NS)) after Bonferroni correction for multiple comparisons). Note the similar causality strength before CFC between nonrecruited and recruited neurons. Data were analyzed by one-tailed Mann–Whitney *U*-test; *U* = 10). Bars show mean ± s.e.m. **d**, Top: Distribution of neuronal recruitment. Bottom: Box plot (median, middle line; 25% and 75% quartiles; whiskers, maximal and minimal values) showing a higher baseline firing rate in future recruited neurons (red). Data were analyzed by two-tailed Student’s *t*-test (*t* = 7.27, d.f. = 221, *n* = 143 (nonrecruited) and 80 (future-recruited) neurons, ***P = 0.0002). Recordings were acquired in 5- to 6-week-old Sprague–Dawley rats of both sexes.

**Extended Data Fig. 9:**
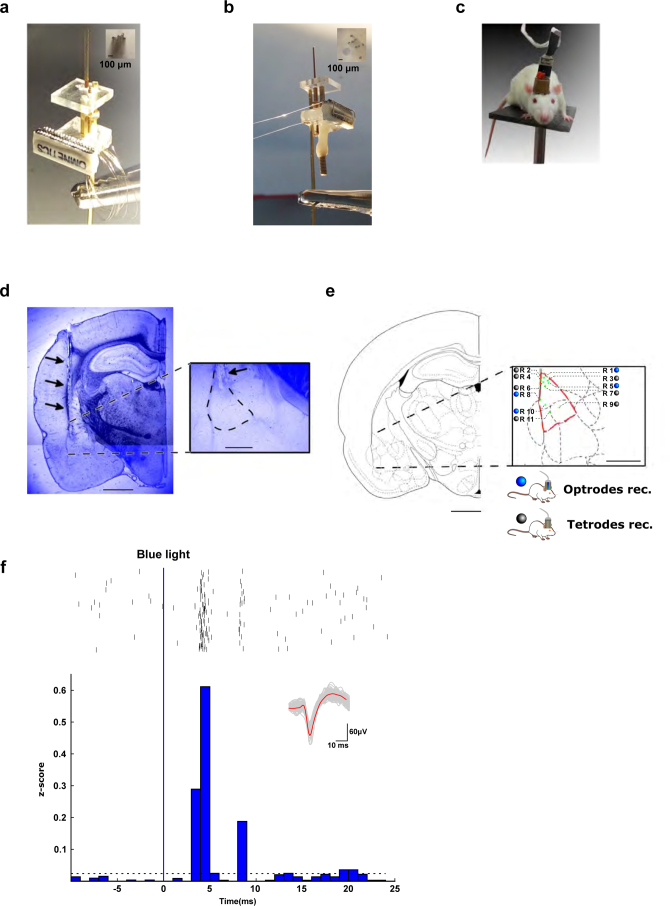
*In vivo* recordings – tetrode implantation. **a**, Microdrive with 8 tetrodes (32 electrodes total) used for *in vivo* electrophysiological recordings in freely-moving rats. **b**, Optical fiber that allows – in addition to recording – stimulation with blue light. **c**, Rat implanted with a microdrive. **d**, DAPI staining of a coronal section of the lateral amygdala (inset: LA, dashed outline) showing the localization of the implanted tetrodes (black arrows). Image shows a representative example of tetrode or optrode placements in 11 rats. Scale bars indicate 2 mm or 0.5 mm (inset). **e**, Localization of all electrode tips, determined post-hoc. Scale bar indicates 1 mm. **f**, Example of Channelrhodopsin-2-expressing neuronal unit responding to a 1-ms blue-light pulse with time-locked spikes following the blue stimulus within a few ms (top, rasterplot data, n = 540 repetitions; bottom, z-score representation with the horizontal dashed line representing 3 standard deviations of baseline activity); inset: spike waveforms of recorded unit.

Contrary to the above ex vivo measurements that can only make cross-comparison changes in synaptic strength between recruited and nonrecruited connections after CFC, in vivo measurements can directly assess any changes induced by CFC longitudinally within the same animal. We thus conducted a series of experiments in behaving animals in which we measured multi-single-unit activity from conditioned stimulus (CS^+^)-responsive LA neurons throughout a 6-h period before and after CFC (Extended Data Fig. 9). After identifying recruited neurons (by their enhanced firing to the CS^+^ after CFC; Fig. 6a), we retrospectively traced back their connectivity levels before CFC. We then used Granger causality analysis of baseline spiking levels to statistically assess changes in functional connectivity between principal neurons (Methods^28,29^). Across all neurons, we found that functional connectivity levels (2.8 ± 4.7% (±s.d.), 78 connections, *n* = 7 rats, *n* = 6,929 possible connections) and the distribution of connectivity motifs both corresponded with the in vitro findings (compare Figs. 6b and 3b), corroborating the comparability between our in vitro and in vivo measurements. Before CFC, functional connectivity levels did not differ between future CFC-recruited and nonrecruited neurons, already suggesting no functional connectivity bias in recruitment (Fig. 6c). Moreover, only after CFC did we find that recruited neurons, in contrast to nonrecruited neurons, exhibit significant increases in functional connectivity (from 0.6 ± 0.3 to 3.7 ± 1.0 artificial units (AU) versus 0.5 ± 0.5 AU to 0.3 ± 0.1; Fig. 6c). We have to acknowledge that the Granger causality approach does not allow us to definitely determine in vivo that local recurrent synaptic connections between recruited/engram neurons are strengthened as a result of fear conditioning, and it is still possible that in vivo coupling could be driven by other factors (for example, distal inputs to LA) rather than local synaptic changes. However, given the correspondence between our ex vivo and in vivo findings, there is compelling evidence that does suggest the later interpretation. Taken together, these findings point to an enhanced local functional connectivity that is caused by, rather than at the origin of, recruitment in the fear memory engram.

Further supporting our previous observations, these in vivo findings also showed that future to-be recruited neurons exhibited significantly higher baseline firing rates before CFC than nonrecruited neurons (Fig. 6d). This finding confirms the notion that intrinsic excitability can predict recruitment as shown previously ex vivo by others^6^ and as we had found in vitro before Hebbian induction (Fig. 4 and Extended Data Fig. 4).

### Optogenetic reactivation of recruited neurons

**Fig. 7:**
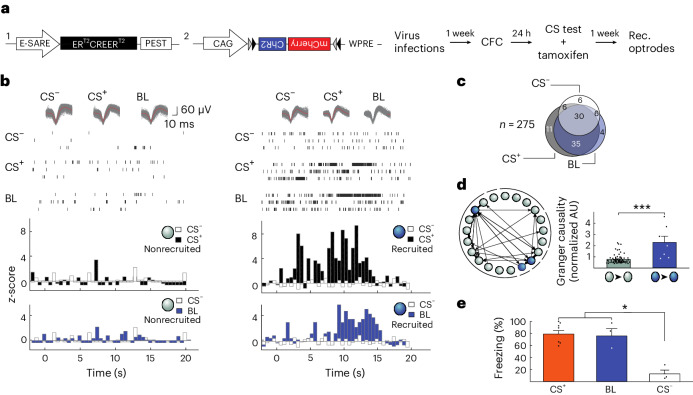
Activating the optogenetically tagged fear memory engram induces freezing behavior. **a**, Viral constructs and the CFC protocol (Methods) of experiments in **b**–**d**. **b**, Examples of neuronal spiking and *z*-scores in response to the CS^+^ (black) and BL (blue). **c**, Venn diagram of neurons responding to the CS^+^, BL and CS^−^(5 kHz tone not paired to the US, see Methods). **d**, Left: Connectivity diagram. Right: Connection strength as a function of recruitment (*n* = 67 (black) and 7 (blue) connections; *U* = 44.5, ***P < 0.001; two-sided Mann–Whitney *U*-test). Bars show mean ± s.e.m. **e**, Freezing levels after CFC in response to the CS^+^, BL and CS^−^ (mixed-model ANOVA, *F*_2,4_ = 21.15, *P = 0.0075 and *P = 0.0104 or 0.0196 for CS^–^ versus CS^+^ or BL, respectively, after Bonferroni correction); *n* = 7 (CS^+^) or *n* = 3 (BL and CS^–^) rats. Bars show mean + s.e.m. Recordings were acquired in 5- to 6-week-old Sprague–Dawley rats of both sexes.

**Extended Data Fig. 10:**
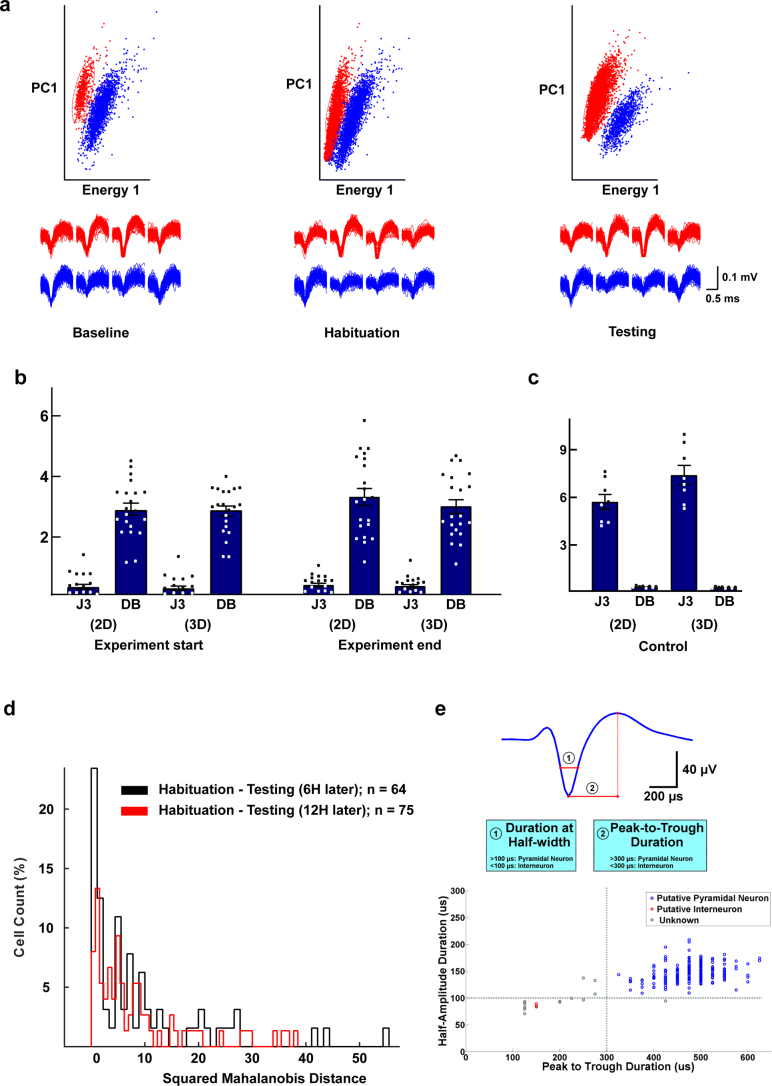
*In vivo* recordings – tracking neurons over time. **a**, Two isolated (red and blue) units originating from the same tetrode, as determined by (top) principal component (PC) and energy analysis. The units were recorded throughout baseline (left), habituation (middle) and testing (right). Bottom, representative waveforms of the recorded neurons. **b**, Cluster stability was assessed by measuring the J3 and Davies-Bouldin (DB) statistics in 2-dimension (2D) principal component space and 3-dimension (3D) principal component space, at the start and end of experiments (see M&M). Bars are means ± sd; n = 21 clusters. **c**, As negative controls to Fig. b), J3 and DB statistics were calculated from eight arbitrary clusters of similar shape and size that were defined from the central spheroid of principal component space (*that is* noise)^25^. Bars are means ± sd; n = 8. **d**, To ensure that the same neuron was recorded over multiple sessions, we quantified the squared Mahalanobis distance, discarding neurons with unstable values across sessions. **e**, Extracellular waveform used as a criterion for distinguishing between interneurons and pyramidal neurons based on the clusters obtained by plotting ‘half-amplitude duration’ against ‘peak to trough duration’.

Our ex vivo and in vivo findings are thus consistent with an organization of neuronal ensembles in the LA through which signal propagation is enhanced after fear learning as a result of potentiation of local connections between recruited neurons. However, ex vivo, we used an activity reporter gene, whereas in vivo, we used CS^+^ responsiveness to identify neuronal recruitment. To confirm that these methods identify the same populations of neuronal ensembles, we developed an in vivo protocol that more closely matched our ex vivo approach. We therefore replaced dGFP with a stable tamoxifen-inducible optogenetic Channelrhodopsin-2 (ChR2) tag under the same *Arc* promoter E-SARE^25^ that we used in the ex vivo experiments. With tamoxifen, 24 h after CFC, we opened a time window to initiate ChR2 expression by CS^+^ recall (Fig. 7a and Methods). After 1 week of ChR2 expression (Extended Data Fig. 8c) and LA optrode implantation (Extended Data Fig. 9a–e), we found that most identified principal LA neurons (Extended Data Fig. 10e) that responded to the CS^+^ also responded to the blue light (BL) (65 out of 82; Fig. 7b,c, Extended Data Fig. 9f and Supplementary Note 6). This 79% overlap is close to the percentage of activated neurons that are typically labeled with our E-SARE approach^25^. Having established this overlap, we used Granger causality analysis and found significantly stronger causal connectivity levels between recruited neurons (BL responsive, 2.3 ± 0.4 AU, *n* = 4) than between nonrecruited neurons (0.8 ± 0.1 AU, *n* = 67; Fig. 7d) also in BL-responsive neuronal ensembles. Moreover, behaviorally, reactivation of these neurons, which had been active previously during fear memory recall, by BL fully recapitulated the freezing levels as seen after presentation of the CS^+^ (75% for BL and 79% for CS^+^; Fig. 7e). Together, these findings indicate that these tagged neurons (and by inference dGFP-expressing neurons) identify the same recruited ensembles in ex vivo and in vivo measurements, further supporting the comparability between these different approaches.

## Discussion

Here, we examined the extent of local connectivity among LA neurons and how these synaptic connections are modulated by fear learning. We found that the connectivity levels throughout the in vitro (2.1%), ex vivo (1.9%) and in vivo (2.8%) preparations, together with the in vitro- and in vivo-identified connectivity motifs, all converge onto the identification of an autoassociative, sparsely connected excitatory network in the rat LA with self-propagating and filtering characteristics that, in basic configuration, permit transmission across the LA of repetitive stimulations and, after fear learning, may allow for propagation of single stimuli through synaptic potentiation between locally recruited LA neurons. Although based on a limited number of observations, due to sparse connectivity levels, together, these findings reposition the LA from a passive relay station into an active hub where synaptic plasticity strengthens LA–LA connections within neuronal ensembles following fear learning. Thus, fear memory encoding involves not only the recruitment of intrinsically more highly excitable LA neurons and the potentiation of their external afferents but also stronger binding of these neurons together in a local network that, after fear learning, can promote signal processing across the LA.

Recent in vivo findings in the visual cortex have shown that Hebbian paradigms can artificially imprint neuronal ensembles for coactivation^30^, and this was shown to affect high-performance behavioral discrimination tasks^31^. Meanwhile, the synaptic and circuit mechanisms by which this happens are still unclear and remain an area of intense investigation^9^. In the mouse basolateral amygdala, different neuronal ensembles can encode distinct behavioral states^32,33^ or learned associations with distinct aversive events^34^ or separated by different time intervals^35^. However, following coactivated recall, integration between individual memories may occur together with a coallocation of partially overlapping neuronal ensembles. The sparse connectivity that we found in the LA would allow storing such multiple memories within and between neuronal ensembles by strengthening of existing connections or the formation of additional connections (see also Lisman et al.^7^). Furthermore, the autoassociative features of this network could permit single neurons to trigger reactivation of connected neurons all or not part of multiple ensembles, leading to pattern completion^9,36^, and contribute to the stabilization of memories^7^ and/or the integration of individually formed memories, for example, such as occurring during second-order fear conditioning^37^.

Recently, in the primate amygdala, the importance of timing and the order of spiking activity for memory encoding was shown, suggesting that even different sequences could encode stimuli of opposite valence^38^. The circuit mechanisms that can generate such reliable sequences, however, are yet to be demonstrated and will require techniques of high spatiotemporal resolution. The present combination of electrophysiological recordings allows such high-resolution measurements and provides functional insights in signal processing and memory formation by showing intranuclear plasticity within the LA, a most primitive cortex. It brings forth a mechanism that places plasticity within neuronal ensembles at the heart of fear memory encoding. The LA could therefore serve as an important model system to study learning and memory through local plasticity within other autoassociative cortical networks.

## Methods

### Animals

We used in-house-bred Wistar (14–19 days old; Ecole Polytechnique Fédérale de Lausanne) and Sprague–Dawley (4–6 weeks old; Center for Psychiatric Neuroscience) rats of both sexes. We found no differences in electrophysiological measurements between different ages, strains (see Figs. 2–4 versus Fig. 5) or sexes, so all animals were pooled together (see also Supplementary Note 1). Animals were housed at room temperature (~20 °C) and placed under a 12-h light/12-h dark cycle, with behavioral experiments performed during the light cycle. All animal handling procedures were approved by the Veterinary Service of the Canton of Vaud (authorizations VD2745 and VD3205).

### Whole-cell recordings on acute brain slices

Animals were decapitated, and their brains were swiftly extracted and placed in chilled artificial cerebrospinal fluid (ACSF). The ACSF slicing solution was saturated with oxycarbon (95% O_2_ and 5% CO_2_) at pH 7.4 and contained 110 mM sucrose, 60 mM NaCl, 28 mM NaHCO_3_, 3 mM KCl, 1.25 mM NaH_2_PO_4_, 7 mM MgSO_4_, 0.5 mM CaCl_2_ and 5 mM d-glucose (Sigma-Aldrich). Acute, horizontal 400-μm-thick rat brain slices were cut between −8.6 mm and −7.6 mm depth from bregma^39^ using a vibratome (Compresstome VF-200, Precisionary Instruments); the presence of external capsule fibers and the beginning of the lateral ventricle were used as landmarks. After slicing, each of the usually obtained four slices was transferred on a nylon grid in a beaker filled with extracellular oxygenated ACSF solution (described below), with a recovery period of at least 1 h at room temperature before being transferred to the recording chamber. Under hyperexcitable conditions, to study epileptiform bursting activity, the KCl concentration was increased to 5 mM, and bicuculline-methiodide (20 µM; Sigma-Aldrich) was added to block GABA_A_ receptors.

A semiautomated 12-patch-clamp setup^40^ (Fig. 1) was used to allow multiple-patch-clamp recordings. Cells were visualized by infrared differential interference contrast video microscopy using a VX55 camera (TILL Photonics) mounted on an upright BX51WI microscope equipped with an Olympus U-RFL-T lamp housing a 100-W mercury burner (Olympus Corporation). A group of up to 12 cells were selected for the electrophysiological recordings based on their morphology (pyramidal shaped) and, where applicable, their fluorescence. The identity of these cells was further confirmed following the injection of square pulses of hyperpolarizing and depolarizing 400-ms currents in 50-pA steps^41,42^ to assess accommodating and nonaccommodating subtypes or interneurons based on their high-frequency (that is, >30 Hz) firing rates. Besides the number of APs resulting from sustained current injection, accommodating and nonaccommodating neurons could be separated further by the delay required to observe an AP (that is, time-to-spike) following minimal stimulation (400 ms, 20-pA steps) with 111 ± 12 ms for nonaccommodating neurons and 82 ± 8 ms for accommodating neurons. In this manner, we classified nonaccommodating neurons with a time-to-spike of >100 ms. Interneurons were excluded from further experimentation and analyses (see also Supplementary Note 1 for more details).

Electrophysiological data were acquired with a Multiclamp 700B (Molecular Devices) in either current clamp or voltage clamp mode. Data acquisition was performed through an ITC-1600 board (Instrutech) connected to a PC running a custom-written routine (Pulse-Q) under IGOR Pro (Wavemetrics, version 7). Recordings were sampled at 10 kHz, and the recorded signal was filtered with a 5-kHz Bessel filter.

Recording pipettes of 4–10 MΩ were pulled from borosilicate capillary glass (Sutter Instrument; outer diameter: 1.5 mm; inner diameter: 0.86 mm; 7.5 cm length) by a P-97 Flame-Brown Micropipette Puller (Sutter Instrument). The pipettes were filled with an internal solution composed of 135 mM KMeSO_4_, 8 mM NaCl, 10 mM HEPES, 2 mM Mg_2_ATP, 0.3 mM Na_3_-GTP and 1 mg ml^−1^ biocytin (Sigma-Aldrich) with a pH of 7.3 and an osmolarity of 300 mOsm. The ACSF in the recording bath was composed of 118 mM NaCl, 25 mM NaHCO_3_, 10 mM d-glucose, 2.5 mM KCl, 1 mM MgCl_2_, 1.25 mM NaH_2_PO_4_ and 2 mM CaCl_2_ (Sigma-Aldrich) dissolved in deionized water of 18.2 MΩ cm resistivity. ACSF was supplemented with 1 mM l-glutamine (Sigma-Aldrich) to avoid homosynaptic depression^43,44^. Recorded neurons were considered stable in current clamp configuration if their membrane potential was lower than −55 mV and in voltage clamp configuration if less than 200 pA was required to maintain the membrane potential at −70 mV.

### EPSP and EPSC analyses

Evoked EPSPs and EPSCs were analyzed using Mini Analysis software (Synaptosoft). Synaptic delay was measured as the time difference between the peak of the presynaptic AP and the onset of the postsynaptic response. Other criteria used for selecting EPSPs were 100 μV for minimal amplitude and 1 mV^2^ for the minimal area under the EPSP. A duration of 5 ms was used as a baseline, sampled 20 ms before the peak. For EPSCs, the minimal amplitude was 5 pA. Finally, the root mean square of the noise was measured over 0.5 ms at the beginning of each trial, outside of spontaneous or evoked responses, and was used for estimating the parameters of quantal analysis.

### Assessment of connectivity

Once a patch clamp was obtained, a 3-ms, 1- to 4-nA square pulse was injected in each neuron to determine the AP firing threshold. Nine suprathreshold pulses were then delivered with eight pulses at 20 Hz, followed by a recovery pulse 550 ms later. This stimulation pattern was delivered successively to each of the patched neurons and repeated 15 times (Fig. 1c). Following this, an average of the responses to each neuronal activation was plotted, and the traces were assessed for time-locked EPSPs occurring within <5 ms with <2.5-ms jitter^16,45^. The presence of such EPSPs indicated a connection between neurons, which was further subjected to visual inspection that could readily and unequivocally confirm the actual presence of a connection (see examples in Extended Data Fig. 1b). Confirmed connections were then further subjected to quantal analysis or plasticity. LA network connectivity was calculated as the proportion of connections found in a given slice to all possible connections. To calculate the number of possible connections, while excluding autoconnections, we used *n*(*n* – 1), with *n* equal to the number of patched neurons for a given slice.

### Quantal analysis experiments

The Ca^2+^:Mg^2+^ ratio was modified to isolate the synaptic quantum. Lower Ca^2+^ concentrations are characterized by a lower probability to observe an EPSC, thereby facilitating the extraction of the quantal size^46^. The following concentrations were used:

[Ca^2+^] = 2 mM and [Mg^2+^] = 1 mM (initial ACSF solution, as described above),

[Ca^2+^] = 1 mM and [Mg^2+^] = 2 mM and

[Ca^2+^] = 0.5 mM and [Mg^2+^] = 2.5 mM.

EPSCs were measured following the same stimulation pattern (for one trial, 8 + 1 APs delivered at 20 Hz), as described above. At least 30 trials were recorded at 2 mM Ca^2+^, and at least 100 trials were recorded at lower concentrations, and the first response of each trial was used for extracting the quantal parameters. The intertrial interval was at least 8 s. Stability of the response was tested by comparing the average response value for the first trial with the last ten trials. Connections that had over 30% variability were excluded from the analyses.

To estimate quantal size, we then identified EPSC and EPSP peaks from individual traces and fed these into a simple binomial model based on earlier observations that multiple release sites on cortical synapses share similar release probabilities^47,48^. Therefore, EPSC and EPSP amplitudes were drawn from a simple binomial distribution with a given number of release sites (*n*), release probability (*p*) and quantal size (*q*)^49^. The mean and standard deviation of this simple binomial distribution are given by

$${{\mathrm{mean}}}={npq}$$

$${\mathrm{standard}}\,{\mathrm{deviation}}\,=q\sqrt{[{np}\left(1-p\right)]}.$$

Extraction of quantal parameters was performed using a custom MATLAB script developed by Hardingham et al. to successfully describe the quantal content of the cortical layer 2/3 synapse using the above simple binomial model^48,50^. This model also uses the value for the recorded noise to better estimate *n*, *p* and *q*. In addition, the maximum likelihood method^51^ was used to fit the acquired data (fminsearch function from MATLAB’s Optimization Toolbox), starting with the lowest estimate for *n* and increasing until a maximum of arbitrarily defined 14 release sites was reached (if the model reached the maximum number of release sites, the resulting fit was discarded). For increased accuracy, the data were fitted against ten different starting points in the parameter space. Finally, the resulting fit was tested against simulated datasets sharing the same parameters using the Monte Carlo simulation and the *χ*^2^ test.

The binomial model has the following parameters:

*v* = amplitude of EPSC or EPSP;

*v*_0_ = offset, assumed to be added to all EPSCs or EPSPs;

*σ*_noise_ = standard deviation of noise, assumed Gaussian;

*n* = number of release sites;

*q* = quantal size;

*p* = release probability at each release site;

*p*_stim_ = probability that stimulation results in an AP that reaches the release sites (one in our case);

m*σ*_q_ = quantal variance, equals standard deviation on first peak in absence of noise; and

*σ*_*m*_ = variance affecting the *m*th peak, where *m* ranges from 0 to *n*.

For type I quantal variance, ${\sigma }_{{\rm{m}}}^{2}={\sigma }_{{\rm{noise}}}^{2}+{\rm{m}}{\sigma }_{{\rm{q}}}^{2}$,

for ‘flat’ quantal variance, *σ*_m_ = *σ*_noise_ for *m* = 0, and ${\sigma }_{{\rm{m}}}^{2}={\sigma }_{{\rm{noise}}}^{2}+{\sigma }_{{\rm{q}}}^{2}$ for m > 0.

The probability density function *f*(*v*) for estimating the EPSP or EPSC amplitude (*v*), as it was used in the original MATLAB script^48,50^, was the following:

$$\begin{array}{l}f\left(v\right)={p}_{{{\mathrm{stim}}}}\mathop{\sum }\limits_{k=1}^{n}\frac{n!}{m!\left(n-m\right)!}{p}^{m}{(1-p)}^{n-m}\frac{1}{{\sigma }_{k}\sqrt{2\pi }}\exp \left(-\frac{{(v-{v}_{0}-,q)}^{2}}{2{\sigma }_{k}^{2}}\right)\\\qquad\quad+\left(1-{p}_{{{\mathrm{stim}}}}\right)\frac{1}{{\sigma }_{{{\mathrm{noise}}}}\sqrt{2\pi }}\exp \left(-\frac{{\left(v-{v}_{0}\right)}^{2}}{2{\sigma }_{{{\mathrm{noise}}}}^{2}}\right).\end{array}$$

### Plasticity protocol

To trigger pre- and postsynaptic APs, a square pulse stimulus was used (1–2 nA, 3 ms) in a train of ten stimuli at 30 Hz repeated 15 times (intertrial interval of 10 s), with the presynaptic potential leading the postsynaptic potential by 5–10 ms (refs. ^24,52^). For testing whether the long-term potentiation-inducing protocol led to changes in EPSP amplitude, the same protocol was used as for assessing connectivity (eight pulses at 20 Hz, followed by a recovery pulse 550 ms later), which was applied every 30 s for up to 1 h, with potentiation typically lasting 20–30 min, that is, until deterioration of the multiple patched cells.

### Surgery

All surgeries were performed under aseptic conditions with isoflurane anesthesia (5% initially and then 2% for maintenance) on a stereotaxic frame (David Kopf Instruments). Animals were kept on a heating pad throughout the duration and recovery from surgery. The animal’s scalp was opened to expose the skull, which was then cleaned with 3% H_2_O_2_. For both virus injections and in vivo electrophysiology, bilateral holes were drilled at −3.00 mm (anterior–posterior) and ±5.15 mm (medial–lateral) relative to bregma. If the bregma-to-lambda distance was less than 8.72 mm (reference for adult^39^), these coordinates were proportionally adjusted.

### Viral vector and virus injection and infection

To fluorescently tag recently activated memory-participating neurons in the LA, we expressed dGFP (a fusion of Venus with an mODC PEST sequence with a half-life of 2 h (ref. ^53^); excitation peak: 515 nm; emission peak: 528 nm) under a modified minimal *Arc* promoter downstream of a synthetic E-SARE^25^.

#### Fluorescent tagging of recruited neurons ex vivo

We used an AAV 2/1 vector to express the E-SARE-driven dGFP. In addition to E-SARE–dGFP, the AAV contained a second cassette that expressed an RFP as an infection marker (RFP635; excitation peak: 588 nm; emission peak: 635 nm) under the constitutive enhanced phosphoglycerate kinase-1 (*Pgk1*) promoter, with a woodchuck hepatitis post-transcriptional regulatory element^54^.

Rats were infused bilaterally in the LA with the AAV at 4 weeks of age under isoflurane anesthesia and semisterile conditions. The bregma coordinates used were anterior–posterior −3.00 mm, medial–lateral ±5.15 mm and dorsal–ventral −7.8 mm. The injector consisted of a glass pipette containing 1.2 μl of AAV at a titer of 1.4 × 10^13^ genomic copies per ml. One microliter was lowered to a depth of 7.8 mm, and virus was infused over 10 min. The pipette was left in place for an additional 3 min to allow viral diffusion^55^.

#### Optogenetic tagging of recruited neurons in vivo

For activity-dependent optogenetic tagging following fear memory recall, we used a dual AAV system, in which the expression of double-floxed E-SARE-ChR2, delivered by one AAV, was controlled by tamoxifen-inducible recombinase ER^T2^CreER^T2^ delivered by another AAV^56^. As previously shown for tamoxifen-based gene activation^25^, to induce ChR2 expression, tamoxifen (10 mg) was administered by gavage 8 h before fear testing, as for mice^25^. This time window coincides with peak tamoxifen metabolism into 4-hydroxytamoxifen in rats^57^. Thus, 1 day after CFC, rats were administered tamoxifen and exposed only to the CS^+^ 8 h later to induce stable expression of ChR2 in recruited neurons following CFC recall. ChR2 expression was assessed by BL responses 1 week after CFC recall.

### Microdrive implantation

Microdrives were built in-house and were implanted according to the same coordinates as used for the virus injections. They included eight tetrodes for a total of 32 channels. The tetrodes were assembled from nichrome wire of 25 μm in diameter (STABLOHM 675 California fine wire), which was insulated with heavy Formvar^58^. Tetrodes were mounted on the microdrive on a copper screw with a 270-μm step. For the optogenetic experiments, an optical fiber was mounted 200 μm away from the tetrode bundle.

To stably anchor the implantation, four fixation points surrounding the microdrive were created to each harbor a small bone screw, two of which were attached to a ground wire. A dental cement layer was used to secure the screws to the skull. The microdrive was lowered to a depth of 6.8 mm. The space between the electrodes and the skull was filled with softened paraffin. An additional layer of dental cement firmly attached the microdrive to the skull. Finally, a copper screen was fitted around the implanted microdrive as a partial Faraday cage to reduce noise during the recordings.

### Electrophysiology in vivo

Electrodes were connected to a headstage (Plexon) containing 32 unity-gain operational amplifiers. The headstage was connected to a 32-channel computer-controlled preamplifier (with a gain of 1,000 and bandpass filter from 400 Hz to 7 kHz, Plexon). Neuronal activity was digitized at 40 kHz bandpass filtered from 250 Hz to 8 kHz and isolated by time–amplitude window discrimination and template matching using a multichannel acquisition processor system (Plexon).

During fear conditioning, spike waveforms and associated time stamps were recorded. For analysis, the artifact waveforms were removed, and the spike waveform minima were aligned using Offline Sorter 4.0 software (Plexon). Principal component scores were calculated for unsorted waveforms and plotted on three-dimensional principal component space, and clusters containing similar valid waveforms were manually defined (based on principal component and waveform feature spaces; Extended Data Fig. 10). A group of waveforms was considered to originate from a single neuron if it was defined as a discrete cluster in principal component space that was distinct from clusters for other units and if it displayed a clear refractory period (1.2 ms) in autocorrelograms^59,60^. Template waveforms were then calculated for well-separated clusters and stored for further analysis in MATLAB and to track neurons over time. To ensure that the same neuron was recorded over multiple sessions (6 h or more), we quantified the squared Mahalanobis distance, discarding neurons with unstable values. For further confirmation, we also measured cluster stability across recording sessions using J3 and Davies–Bouldin statistics^59,60^.

For each isolated unit and for each experiment, neuronal spikes were plotted as a raster plot of time stamps relative to stimulus exposure (CS^+^, CS^–^ and BL; *t* = 0). Spike counts were binned in 50-ms bins (Fig. 6) or 0.5-s bins (Fig. 7) and normalized to a 500-ms (Fig. 6) or 5-s (Fig. 7) baseline average to obtain a *z*-score. Neurons were considered responsive to a stimulus if the *z*-score value of their activity crossed the significance level (3 s.d. compared to prestimulus baseline)^59^. In particular, for optogenetic experiments, neurons were considered BL sensitive if they responded with time-locked (<5-ms jitter) millisecond precision^61^ (Extended Data Fig. 9f).

### Behavior: auditory CFC

To fear condition the rats to a tone (the CS), animals were placed in a fear conditioning box, which included a metal grid for scrambled shock delivery (0.45 mA over 2 s) to the feet and a clear Plexiglass top for camera recording. The delivery of the tone (12 kHz, 250-ms blips presented at 1 Hz over 20 s) and the shock as the unconditioned stimulus (US) were controlled by MedPC IV software. The stimuli (CS and/or US) were presented at random intervals (1–3 min). Auditory fear memory recall was tested in a different context, the testing cage, which was a hexagonal cardboard box with wooden bedding. The conditioning and test boxes were cleaned with a solution containing 70% ethanol after each session.

Animals were randomly assigned to three independent groups: homecage, CS/US paired and CS/US unpaired. The homecage group was not exposed to any aspect of the behavioral experiment (including surgery, habituation and handling). Following a 3-day recovery period from surgery, the rats from the unpaired and paired CS/US groups were handled and habituated to the conditioning cage and the testing cage in 5-min sessions once per day for 3 days. One day after the last habituation session, the animals were placed in the conditioning chamber. After a delay of 5 min, the paired group received three paired co-terminating presentations of the CS–US over 3–9 min. The unpaired control group received three consecutive US presentations, followed by three CS presentations. Twenty-four hours after fear memory acquisition, rats from the paired and unpaired groups received three presentations of the CS in the testing cage and were then returned to their home cages. After a delay of 90 min to achieve optimal dGFP expression, the animals were killed either by decapitation (for use for electrophysiology) or were deeply anesthetized with 4% isoflurane and perfused with phosphate buffer (PB; pH 7.4) and formaldehyde (4% in PB; fixation for confocal microscopy). Animals from the homecage group were killed following the same procedures. For the in vivo optogenetics experiments (Fig. 7b–d), a second tone (CS^−^; 5 kHz, continuous tone over 20 s) was presented that was never paired to the US, and we infused AAVs to make tamoxifen-inducible expression of ChR possible (see above). One week later, animals were subjected to CFC, and fear memory was recalled 24 h after learning in the presence of tamoxifen. After one additional week to allow ChR to be expressed, fear was recalled again, and neuronal responses to the CS^+^, CS^–^ and BL were recorded. An additional group of animals (*n* = 4) was used for visualizing the fluorescent reporter ChR2 by confocal microscopy (Extended Data Fig. 8c). For the short-term memory in vivo experiments (Fig. 6), CS and US association and subsequent CS memory testing were separated by a 6-h interval^21^. We chose this shorter time interval to maximize the probability of recording from the same neurons reliably at the beginning and end of the experiment.

A FT200EMT optical fiber (Thorlabs) was mounted 200 μm adjacent to the tetrode bundle, with a laser (Dream Lasers) ensuring BL delivery at 473 nm, with a power at source of 50 mW and ~15 mW at the tip of the optical fiber. BL was delivered with a pulse width of 2 ms, either as a single pulse or at 20-Hz frequency trains.

Rats were considered as freezing to a stimulus (CS or BL) if no movement was detected for at least 2 s; the sum of the freezing bouts was then expressed as a percentage of the stimulus presentation. Freezing was assessed by an experimenter blind to the experimental conditions.

### Immunohistochemistry and confocal image acquisition

For confocal microscopy experiments, following perfusion, brains were extracted and postfixed for 2 days in 4% formaldehyde and cryoprotected in 30% sucrose for an addition 2 days. Horizontal sections (50 µm thick) were cut on a MICROM HM 440E microtome (GMI).

To visualize inhibitory neurons, nonspecific binding sites on free-floating sections were blocked with 2% normal horse serum (Jackson Immuno Research Laboratories) in 0.1 M PB (pH 7.4) supplemented with 0.3% Triton X-100 (Sigma-Aldrich) and sodium azide (1 g l^–1^; Sigma-Aldrich) for 1 h at room temperature. Sections were then incubated with mouse primary anti-GAD67 (1:2,500; MAB5406, Merck Millipore) in blocking buffer for 48 h at 4 °C. To visualize antibody–antigen complexes, an AlexaFluor 405-conjugated goat anti-mouse antibody (1:300; A31553, Life Technologies) was applied in PB (with 0.3% Triton X-100 and sodium azide; Sigma-Aldrich) for 1 h at room temperature. Sections were then mounted with Vectashield (Vectorlabs) and stored at 4 °C until assessment by microscopy.

Fluorescence of both dGFP and mCherry following viral expression was sufficiently strong to be visualized directly.

Images were acquired using a Zeiss LSM 780 Quasar Confocal Microscope (Zeiss). Three lasers were used to excite at 405, 488 and 561 nm (diode, argon and diode-pumped solid-state lasers, respectively) at 2–3% power. Zen 2012 software was used to control the acquisition parameters of the LSM 780. Constant parameters included a pixel depth at 16 bit, filtering the average of two values and scanning unidirectionally and in ‘Line’ mode.

An ImageJ script was used to automatically detect fluorescence thresholds and count cell bodies. Cutoff parameters were used to minimize the inclusion of false positives. Exclusion criteria included cell size (minimal cutoff of 45 μm^2^ neuron body area) and fluorescence intensity (minimal cutoff of 15,000 average pixel value for a given channel out of a maximal 2 (ref. ^16^) or 65,536 for saturated pixels).

### Statistics

All statistical analyses were conducted with GraphPad Prism 9 and R4.2 (ref. ^62^). Sample sizes were determined online based on mean difference and standard deviation (http://www.biomath.info; Center for Biomathematics, Department of Pediatrics at Columbia University Medical Center). When comparing populations, data were first tested for normality with either the Kolmogorov–Smirnov test or, when the sample data size was less than 50, with the Shapiro–Wilk test and for homogeneity of variance using Bartlett’s test. If the data did not deviate significantly from normal distribution and the variances were homogenous, independent samples *t*-tests, paired *t*-tests or ANOVAs were used. Results that were found to deviate significantly from the normal distributions and/or whose variance was not homogenous were analyzed with appropriate nonparametric tests (see below for a list of tests used). When multiple comparisons occurred, the tests were Bonferroni corrected.

When the Kolmogorov–Smirnov test was used to compare differences between two distributions, the cumulative frequency of each distribution was normalized to its maximal value.

For the RM-ANOVA, data were tested for sphericity using the Mauchly test, and to correct for departures from sphericity, the Greenhouse–Geisser and Huynh–Feldt corrections were applied^63–65^. Where applicable, statistical tests were two tailed. For cross-correlations, the corrplot function in MATLAB (MathWorks, 9.6) was used, with a Pearson test for linear correlations and a Spearman test for nonlinear correlations (resulting in their respective correlation coefficients, *r* and *ρ*).

The frequency of observed connectivity motifs was compared to their respective frequency in a simulated network (100,000 Monte Carlo simulations taking into account real cell positions and random distance-dependent connectivity based on Fig. 3a; see a detailed explanation in Supplementary Note 3).

The following statistical tests and variables (degrees of freedom are shown as underscore numbers) were used:

- Student’s *t*-test uses the *t* variable.
- ANOVA uses the *F* variable.
- Wilcoxon signed-rank test uses the *W* variable.
- Mann–Whitney *U*-test uses the *U* variable.
- Kolmogorov–Smirnov test uses the *D* variable.
- Monte Carlo simulation for network connectivity: *n* = 100,000 simulations.

For neuronal activity represented as *z*-scores, when assessing stimulus response, a poststimulus response was considered significant when its value was greater than 3 s.d. of the prestimulus baseline (see above).

### Granger causality analysis

To determine connectivity (positive relationship with *P* < 0.001) and connection strength (Granger causality statistic) between two recorded neurons in vivo, we used Weiner–Granger vector autoregressive causality analysis as implemented in the multivariate Granger causality toolbox^28,29^. Time stamps recorded from each neuron were converted into a continuous signal by binning in 1-ms increments and convolving the resulting signal with a half-Gaussian filter (5-ms width). This analysis was performed on spike train data gathered over a period of 20 min outside of stimulus exposure. Stationarity was checked and confirmed for all models by determining whether the spectral radius of the estimated full model was less than 1 (ref. ^29^). Model order was derived by using Bayesian information criteria, and the vector autoregressive model parameters were determined accordingly. Subsequently, time domain-conditional Granger causality values were calculated for each neuron pair. Causal density was taken as the mean pairwise-conditional causality and was subsequently normalized to the entire dataset^29^. Because the current model assessed Granger causality between presynaptic and postsynaptic spikes, it could only infer excitatory but not inhibitory connections (Granger causality of presynaptic spike to postsynaptic silence).

Granger causality analysis was always performed on baseline data recorded from the animal at rest (either before or after fear conditioning) to prevent false-positive connections resulting from stimulus-evoked activity. Furthermore, we did not estimate connectivity (only connection strength) after fear conditioning because CFC increased the number of positive connections (*P* < 0.001). However, CFC did not affect connection strength (Granger causality statistics), as assessed in Fig. 6c where connection strength increased only in the recruited connections but not in the nonrecruited connections. Whether neurons were recruited or not was assessed after fear conditioning.

### Reporting summary

Further information on research design is available in the Nature Portfolio Reporting Summary linked to this article.

## Online content

Any methods, additional references, Nature Portfolio reporting summaries, source data, extended data, supplementary information, acknowledgements, peer review information; details of author contributions and competing interests; and statements of data and code availability are available at 10.1038/s41593-024-01676-6.

### Supplementary information

Supplementary Information Supplementary Notes 1–7 and References.

Reporting Summary

## Data Availability

All data are available in the main text or the supplementary information available at Zenodo at 10.5281/zenodo.10890959 (ref. ^66^).

## Code availability

No custom software code was used to analyze the data.
