# Supplementary material for: Fear learning induces synaptic potentiation between engram neurons in the rat lateral amygdala
Source: Nat Neurosci. 2024 Jun 13;27(7):1309–17. doi: 10.1038/s41593-024-01676-6 (PMC11239494; doi:10.1038/s41593-024-01676-6)
Supplement: Supplementary file 1 — Supplementary Notes 1–7 and References. [file 41593_2024_1676_MOESM1_ESM.pdf]

nature neuroscience

Supplementary information

https://doi.org/10.1038/s41593-024-01676-6

Fear learning induces synaptic potentiation between engram neurons in the rat lateral amygdala

In the format provided by the authors and unedited

Supplementary Materials for

Fear learning induces synaptic potentiation between engram neurons in the rat lateral amygdala

Marios Abatis1, Rodrigo Perin2, Ruifang Niu1, Erwin van den Burg1, Chloe Hegoburu1, Ryang Kim3, Michiko Okamura3, Haruhiko Bito3, Henry Markram2, Ron Stoop1*.

Correspondence to: ron.stoop@unil.ch

This PDF file includes:

Supplementary Notes 1-7

References 67-75

Supplementary Notes

Supplementary Note 1: Electrophysiological characterization of neurons and connections and their comparability across different ages, strains and sexes.

For the *in vitro* experiments we based our selection of pyramidal neurons on soma shape and action potential firing rates of < 30 Hz (Extended Data Fig. 1a). On average, pyramidally identified neurons exhibited a membrane resistance of 300 ± 175 MΩ, capacitance of 200 ± 92 pF, and average holding current of -8 ± 32 pA (± s.d.) for voltage-clamp at -70 mV (n = 60). Importantly, in all our recordings (n = 89 - 16 double – 1 triple = 71), only thus-selected neurons turned out to make excitatory connections with postsynaptic targets, thereby validating our selection criteria to correctly identify pyramidal neurons (and discard potential interneurons as targets, Figs. 1&2 & Extended Data Fig 1). We therefore believe that, based on these criteria, any subsequently identified targets of these neurons can indeed also be classified as excitatory pyramidal neurons.

We also identified 21 putative interneurons in our *in vitro* recordings, based on high-frequency spiking patterns (52 ± 24 Hz, ± SD; Extended Fig. 1a) and high membrane resistance (in the range of 1 GΩ). Of those that made connections to postsynaptic targets (10 in total), all of these connections turned out to be inhibitory, thereby validating our criteria for identifying inhibitory interneurons (Extended Data Figs. 1f,g).

The proportion of electrophysiologically-characterized pyramidal neurons (96%) and interneurons (4%) in the lateral amygdala closely corresponds to what has been previously described in the literature for the rat lateral amygdala[67]. For experiments performed on

virus-injected animals *ex vivo* (Fig. 5b-e), we found 14 interneurons and 4 inhibitory $Arc^+ \rightarrow Arc^-$ connections (Extended Data Fig. 1f,g).

Throughout the experiments, we found EPSPs of similar amplitudes in younger naive rats (uEPSP1: 1 ± 1 mV or all uEPSPs: 0.9 ± 0.7 mV (n = 82), Figs. 2-4) and older conditioned and non-conditioned rats used for *ex vivo* studies (pooled together: uEPSP1: 1.2 ± 1.0 mV or all uEPSPs 0.9 ± 0.6 mV (n = 14), Fig. 5). Connectivity, although different *ex vivo* between recruited (GFP+→GFP+) and non-recruited (GFP-→GFP-) connections (Extended Data Fig. 7b), was similar overall at ~2% between younger and older animals.

We did not observe any difference in connectivity between male and female rats (Welch's t-test, W = 123, $P > 0.05$, n = 42 males and 18 females). Connectivity was similar among hemispheres (χ^2 goodness-of-fit test, $\chi^2 = 2$, df = 1, $P > 0.05$) and within slices sampled at different levels of the dorso-ventral axis (χ^2 goodness-of-fit test, $\chi^2 = 5.1$, df = 2, $P > 0.05$). Furthermore, there was no clear distribution of electrophysiological phenotypes. Similarly, connections were spread throughout the LA's rostro-caudal plane.

In vivo we found a connectivity of 2.8±4.7% (78 out of 6929 possible connections; ±s.d.) compared to a connectivity on naïve acute slices of 2.1±0.1% connectivity (±s.e.m.; 89 observed out of 4157 possible connections).

Increases in synaptic or connectivity strength were also comparable across different approaches: *In vitro*, following Hebbian induction in 14-19d Wistar rats we found increases from 1.1 to 1.5 mV, *ex vivo* in 5-6 week SD rats from 0.6 to 1.8 mV and *in vivo* in 5-6 week SD rats a similar doubling to tripling of connectivity strength as assessed by Granger Causality analysis.

Supplementary Note 2: Estimation of the average number of inputs received by a single neuron

The rat LA is estimated to include ~57,000 excitatory neurons in a volume of 1.5 mm^3 [39,68,69]. To estimate the number of inputs that each neuron in the LA receives, we simplified a portion of the LA as a cube of 500 x 500 x 400 μm^3 (as connections were extremely rare beyond 400 µm inter-somatic distance, Fig. 3a); length and width values (500 µm) were slightly greater than height (400 µm) to accommodate for the LA being organized in horizontal modules[70,71]. In this simplified model, each neuron occupies a volume of 20 x 20 x 20 µm, which results in a matrix of 25 x 25 x 20 neurons occupying the 500 x 500 x 400 μm^3 (500/20 x 500/20 x 400/20). In total, this includes 12,500 neurons out of the ~57,000 estimated for the LA. With this defined position for each neuron and with connectivity estimated based on the distance-dependent distribution (Fig. 3a), we estimated that each neuron receives on average ~230 inputs, as modeled with Monte Carlo simulations (n = 100,000) with inputs describing the likelihood of a connection on the basis of inter-somatic distance (see also Supplementary Note 3).

Supplementary Note 3: Random distance-dependent network based on recorded neuron positions (Fig. 3b, 6b)

To assess whether the presence of the different connectivity motifs that we found could be explained by a random network, i.e. by chance, we compared the number of occurrences of these motifs with those that would occur in a simulated randomly-connected network. An example of observed connections is shown in Matrix 1A (example below), with a value of 1 (green) indicating that the neuron on a given row projects to a

neuron in a given column. Conversely, a value of 0 indicates the absence of a connection. For the purpose of the simulation, the Monte Carlo method was used to generate 100,000 data sets based on the real measured inter-somatic distances (for example, see Matrix 1B) with random connections attributed based on the probability to find a connection given the distance (see Fig. 3a).

In detail, for each simulation and for each slice, a table was composed depicting the probability to observe a connection (Matrix 1C) based on the measured inter-somatic distances (Matrix 1B) for that slice and the connection probability distribution (see results; Fig. 3a). This probability table was then compared to a randomly generated probability between 0 and 1, drawn from a uniform random distribution (Matrix 1D, generated with Matlab's *rand* function, with a controlled random number generator seed). A connection was present in the simulated model if the generated probability (Matrix 1D) was below the estimated probability for a given inter-somatic distance.

A: Connectivity Matrix

	#1	#2	#4	#5	#6	#7	#9	#12
#1		0	0	0	0	0	0	0
#2	1		0	1	0	0	0	0
#4	0	0		0	0	0	0	0
#5	0	0	0		0	0	0	0
#6	0	0	0	0		0	0	0
#7	0	0	0	1	0		0	0
#9	0	0	0	1	0	1		0
#12	0	0	0	1	0	0	0	

B: Inter-somatic distance

#1	#2	#4	#5	#6	#7	#9	#12
	115	139	208	222	278	233	106
115		52	99	111	176	162	103
139	52		75	111	185	199	153
208	99	75		53	128	181	190
222	111	111	53		77	135	178
278	176	185	128	77		111	211
233	162	199	181	135	111		137
106	103	153	190	178	211	137	

C: Probability function on distances

	#1	#2	#4	#5	#6	#7	#9
#1		0.05	0.04	0.03	0.03	0.03	0.03
#2	0.05		0.05	0.06	0.05	0.04	0.04
#4	0.04	0.05		0.06	0.05	0.03	0.03
#5	0.03	0.06	0.06		0.05	0.05	0.04
#6	0.03	0.05	0.05	0.05		0.06	0.05
#7	0.03	0.04	0.03	0.05	0.06		0.05
#9	0.03	0.04	0.03	0.04	0.05	0.05	
#12	0.05	0.06	0.04	0.03	0.04	0.03	0.05

D: Simulated connection found

#1	#2	#4	#5	#6	#7	#9	#12
	0.71	0.25	0.69	0.23	0.58	0.84	0.87
0.90		0.27	0.02	0.86	0.89	0.23	0.27
0.10	0.11		0.39	0.20	0.15	0.84	0.14
0.84	0.61	0.62		0.04	0.24	0.40	0.89
0.48	0.23	0.01	0.16		0.41	0.91	0.90
0.45	0.09	0.84	0.89	0.09		0.90	0.74
0.14	0.92	0.54	0.07	0.14	0.31		0.06
0.99	0.81	0.00	0.52	0.92	0.08	0.56	

Example of Monte Carlo simulation to find simulated connections based on the determined probability distribution

(**A**): The connectivity matrix indicates whether neurons in each row (#*n*) project to a corresponding neuron in a corresponding column (#*n*), with a successful connection indicated with a one (green) and a lack of connection with a 0. (**B**): The recorded inter-somatic distances for each pair of neurons. (**C**): The probability to observe a connection, based on the connection probability function and the specified inter-somatic distances. (**D**): Randomly generated numbers (between 0 to 1). When the generated number is strictly lower than the probability in C, then a simulated connection is counted. Orange cells: simulated connections.

Reciprocal and feedforward connections occurred only in 2 and 6 simulations out of 100 000 (*i.e.* 0.02 and 0.06 % respectively), respectively, a number that is negligible

compared to the observed 4 reciprocal and 16 feed-forward connections out of 89 connections that we found i.e. 4 and 18 % respectively). It thus appears that in these slices reciprocal and feedforward connections are much more prevailing than expected from randomness, as also found in the somatosensory[16] and visual[18] cortices.

Supplementary Note 4: Bursting signal propagation (Fig. 3c)

In Fig. 3c, our experiments producing epileptogenic activity in the LA served two purposes. Firstly, to extend earlier findings that had raised the hypothesis of an underlying LA network (without showing direct connectivity) and secondly to illustrate an example of LA excitatory network processing. Indeed, regarding the latter, Samson & Paré[71] have shown that puffing glutamate on horizontal brain sections of the guinea pig, containing the LA, activates poly-synaptic sustained excitatory activity lasting up to hundreds of milliseconds that follows a rostral-to-caudal pathway. Similarly, Johnson et al.[37] have shown that stimulating the internal capsule, in coronal sections of the rat brain, induces 'reverberating' – or recurring 'bursting' – activity lasting at least up to 50 ms. Finally, we have shown[19] that by inhibiting $GABA_A$ receptors and increasing potassium concentration to 5 mM, it is possible to induce epileptogenic activity in the LA[19]. We have replicated this last experiment with one notable difference: the isolation of the LA by severing input and output connections (Fig. 3c, left top panel), to ensure the local nature of bursting activity, and thereby the local nature of the connections. Prolonged incubation (30 or more minutes) in such conditions leads to AMPA-receptor-mediated[19] synaptic transmission evidenced by the appearance of bursts (Fig. 3c, top middle panel).

We positioned electrodes as clusters of up to four electrodes at the caudal, medial and rostral edges of the LA and recorded spontaneous epileptogenic bursting activity. We recorded 43 neurons over 6 slices (average of 5.4 ± 4.7 neurons per slice; ± s.d.). On average, recorded bursts lasted 291 ± 239 ms (± s.d.), when considering a burst ranging from the first to the last action potential; when considering the entirety of a burst's hyper-excited duration, measured over the whole depolarization period, then burst duration was measured at 789 ± 140 ms. The maximal delay for burst onset was 30 ± 4 ms (± s.d., 6 slices), as measured between the first and last bursting neuron (Fig. 3c, top right panel). Furthermore, regarding the burst delay, bursting appeared to successively spread from a caudal-to-medial and a caudal-to-rostral pathway (from LA inputs to output regions targeting the central and basolateral amygdala, respectively). Together, these results suggest a progressive filtering function of the LA network as information spreads through it.

Supplementary Note 5: Estimation of the average number of inputs needed to trigger an action potential

We examined, based upon connectivity levels in the LA of convergent connectivity and facilitation, how many of the 230 inputs would be sufficient to trigger postsynaptic spiking in the lateral amygdala[72]. As we assessed that threshold for spike induction by current injections in the LA amounts to 28.0 ± 0.7 mV (sampled from n = 86 out of a total of 637 recorded LA neurons, Fig. 3e), this would require with a uEPSP1 amplitude of 0.36 ± 0.11 mV (n = 36 measured exclusively in convergent motifs) an average of 100.2 ± 22.9 inputs (±s.d.) required to trigger an action potential. By comparison, an average of only 7.3

± 1.9 inputs are required to trigger an action potential in the CA3[73]. However, we have observed that repetitive stimulation leads to synaptic augmentation (as reflected by high amplitudes of uEPSPR of 0.95 ± 0.17 mV, n = 36, measured in convergent motifs only; see also Fig. 3d and Extended Data Fig. 3a that illustrate uEPSPR facilitation across all LA-LA connections), substantially lowering the number of synaptic inputs to 33.6 ± 7.7 to reach action potential threshold. This suggests that during periods of heightened activity, the LA local network can sustain neuronal activity with activation of fewer than of 15% of local input neurons to trigger an action potential.

Supplementary Note 6: Optogenetic tagging

We have tagged neurons active during the recall of the CFC memory by using a dual AAV-system, in which the expression of double-floxed ESARE-ChR2, delivered by one AAV, was controlled by tamoxifen-inducible recombinase $ER^{T2}CreER^{T2}$ delivered by another AAV[56]. Behaviorally, the successful elevated freezing as a result of blue-light presentation reflects the successful reactivation of neurons that are part of the fear memory engram. Tetrode recordings, coupled with optogenetic stimulation, allowed us to identify neurons that responded with a time-locked (<5 ms jitter) response that varied up to 100 ms (Extended Data Fig. 9f). We have chosen jitter, rather than latency-to-respond to reflect tagging because spike onset for LA neurons depends on the amount of current injected and can have a >100ms delay for very low current stimulations[74]. Also, one could expect blue-light, depending on the intrinsic excitability of the neuron under investigation, to produce action potentials with considerably different delay times between different neurons.

Finally, it is likely that not all LA neurons will be infected by the viruses, so that ChR2 may not be expressed in every cell that becomes part of the engram [75]. However, it has been suggested that stimulating a subset of the memory engram can, through pattern completion, restores activity of the entire memory trace (*in silico*[73]; in brain slices[30]). Thus, we include in our analysis all neurons that display a time-locked response to blue-light activation.

<u>Supplementary Note 7: Number of animals used for each experiment</u>

Figure	Group	Number of rats	Number of connections (sample)
2a,b		21 (out of 34)	82
2c,d		7	10
3a		21 (out of 34)	66
3c		20	75
3d	High facilitation	14	29
3d	Stable	18	32
3d	Low facilitation	7	12
3d	Uncategorized (not shown)	7	9
4a		10	20

5b	3 groups	4 rats per group	N/A
5c,d	dGFP+ connections	3	5
5c,d	dGFP- connections	3	5
6a-d		7	74
7a-d		4	78
Extended 1		34	N/A
Extended 2		21 (out of 34)	75
Extended 3		Same as 3d	Same as 3d
Extended 6b	dGFP+ connections	19	6 (out of 107 possible)
Extended 6b	dGFP- connections	19	5 (out of 369 possible)
Extended 8	3 groups	4 rats per group	N/A

References

67. Sah, P., Faber, E. S. L., Lopez De Armentia, M. & Power, J. The amygdaloid complex: anatomy and physiology. *Physiological reviews* **83**, 803–34 (2003).
68. McDonald, A. J. Neurons of the lateral and basolateral amygdaloid nuclei: A golgi study in the rat. *J. Comp. Neurol.* **212**, 293–312 (1982).
69. Pitkänen, A. & Kemppainen, S. Comparison of the distribution of calcium-binding proteins and intrinsic connectivity in the lateral nucleus of the rat, monkey, and human amygdala. *Pharmacology Biochemistry and Behavior* **71**, 369–377 (2002).
70. Samson, R. D., Dumont, E. C. & Paré, D. Feedback inhibition defines transverse processing modules in the lateral amygdala. *The Journal of neuroscience : the official journal of the Society for Neuroscience* **23**, 1966–1973 (2003).
71. Samson, R. D. & Paré, D. A spatially structured network of inhibitory and excitatory connections directs impulse traffic within the lateral amygdala. *Neuroscience* **141**, 1599–609 (2006).
72. Spruston, N. Pyramidal neurons: dendritic structure and synaptic integration. *Nature reviews. Neuroscience* **9**, 206–221 (2008).
73. Guzman, S. J., Schlögl, A., Frotscher, M. & Jonas, P. Synaptic mechanisms of pattern completion in the hippocampal CA3 network. *Science (New York, N.Y.)* **353**, 1117–23 (2016).
74. Washburn, M. & Moises, H. Electrophysiological and morphological properties of rat basolateral amygdaloid neurons in vitro. *J. Neurosci.* **12**, 4066–4079 (1992).

75. Kawashima, T. *et al.* Functional labeling of neurons and their projections using the synthetic activity–dependent promoter E-SARE. *Nat Methods* **10**, 889–895 (2013).
