# Supplementary material for: Fear learning induces synaptic potentiation between engram neurons in the rat lateral amygdala
Source: Nat Neurosci. 2024 Jun 13;27(7):1309–17. doi: 10.1038/s41593-024-01676-6 (PMC11239494; doi:10.1038/s41593-024-01676-6)
Supplement: Supplementary file 2 — Reporting Summary [file 41593_2024_1676_MOESM2_ESM.pdf]

nature portfolio

Corresponding author(s): Ron Stoop

Last updated by author(s): Apr 12, 2024

Reporting Summary

Nature Portfolio wishes to improve the reproducibility of the work that we publish. This form provides structure for consistency and transparency in reporting. For further information on Nature Portfolio policies, see our Editorial Policies and the Editorial Policy Checklist.

Statistics

For all statistical analyses, confirm that the following items are present in the figure legend, table legend, main text, or Methods section.

n/a	Confirmed	
☐	☒	The exact sample size (*n*) for each experimental group/condition, given as a discrete number and unit of measurement
☐	☒	A statement on whether measurements were taken from distinct samples or whether the same sample was measured repeatedly
☐	☒	The statistical test(s) used AND whether they are one- or two-sided<br>*Only common tests should be described solely by name; describe more complex techniques in the Methods section.*
☐	☒	A description of all covariates tested
☐	☒	A description of any assumptions or corrections, such as tests of normality and adjustment for multiple comparisons
☐	☒	A full description of the statistical parameters including central tendency (e.g. means) or other basic estimates (e.g. regression coefficient) AND variation (e.g. standard deviation) or associated estimates of uncertainty (e.g. confidence intervals)
☐	☒	For null hypothesis testing, the test statistic (e.g. *F*, *t*, *r*) with confidence intervals, effect sizes, degrees of freedom and *P* value noted<br>*Give P values as exact values whenever suitable.*
☒	☐	For Bayesian analysis, information on the choice of priors and Markov chain Monte Carlo settings
☒	☐	For hierarchical and complex designs, identification of the appropriate level for tests and full reporting of outcomes
☐	☒	Estimates of effect sizes (e.g. Cohen's *d*, Pearson's *r*), indicating how they were calculated

Our web collection on statistics for biologists contains articles on many of the points above.

Software and code

Policy information about availability of computer code

Data collection	In vitro electrophysiological data were collected with Axon Instruments Clampex (version 11.0) and in vivo data were collected with Plexon USA (version 4.0). Data acquisition for 12 patch clamp recordings was performed through an ITC-1600 board (Instrutech, Germany), connected to a PC running a custom-written routine (Pulse-Q) under IGOR Pro (Wavemetrics, USA, version 7)
Data analysis	Behavioral data were analysed with Matlab 9.6, electrophysiological in vitro data with Axon Instruments Clampfit (version 11.0) and in vivo electrophysiological data with Offline sorter of Plexon, 4.0 USA. Prism Graphpad 9.0 and R 4.2. were used for statistical analyses. For figure 1, we also used Igor 7 and Illustrator CC. Statistical analysis was performed with GraphPad Prism 9 and R4.2.

For manuscripts utilizing custom algorithms or software that are central to the research but not yet described in published literature, software must be made available to editors and reviewers. We strongly encourage code deposition in a community repository (e.g. GitHub). See the Nature Portfolio guidelines for submitting code & software for further information.

Data

Policy information about availability of data

All manuscripts must include a data availability statement. This statement should provide the following information, where applicable:

- Accession codes, unique identifiers, or web links for publicly available datasets
- A description of any restrictions on data availability
- For clinical datasets or third party data, please ensure that the statement adheres to our policy

A statement was included in the manuscript regarding data availability at the end of the material and methods with a reference to DOI 10.5281/zenodo.10890959

Research involving human participants, their data, or biological material

Policy information about studies with human participants or human data. See also policy information about sex, gender (identity/presentation), and sexual orientation and race, ethnicity and racism.

Reporting on sex and gender	N/A
Reporting on race, ethnicity, or other socially relevant groupings	N/A
Population characteristics	N/A
Recruitment	N/A
Ethics oversight	N/A

Note that full information on the approval of the study protocol must also be provided in the manuscript.

Field-specific reporting

Please select the one below that is the best fit for your research. If you are not sure, read the appropriate sections before making your selection.

☒ Life sciences ☐ Behavioural & social sciences ☐ Ecological, evolutionary & environmental sciences

For a reference copy of the document with all sections, see nature.com/documents/nr-reporting-summary-flat.pdf

Life sciences study design

All studies must disclose on these points even when the disclosure is negative.

Sample size	Sample sizes are given for each figure in the legend. Sample sizes were determined online, based on mean difference and standard deviation (http://www.biomath.info; Center for Biomathematics, Department of Pediatrics at Columbia University Medical Center)
Data exclusions	No exclusion of data
Replication	All experimental figures represent pooled data of two to three independent tests
Randomization	Experimental groups were always mixed, such that all treated and experimental animals were represented in independent tests to exclude day-effects. For figures 1-4, this was not relevant, since this concerned longitudinal experiments with no parallel comparisons. For figure 5-7, animals were randomly assigned to different groups and further electrophysiological experiments were conducted independently by another researcher
Blinding	Behavioral data were double checked by two experienced researchers blind to the groups. For figures 1-4 and related supplementary figures, blinding was not relevant, as observations were not assigned to different experimental groups.

Reporting for specific materials, systems and methods

We require information from authors about some types of materials, experimental systems and methods used in many studies. Here, indicate whether each material, system or method listed is relevant to your study. If you are not sure if a list item applies to your research, read the appropriate section before selecting a response.

Materials & experimental systems

n/a	Involved in the study
☐	☒ Antibodies
☒	☐ Eukaryotic cell lines
☒	☐ Palaeontology and archaeology
☐	☒ Animals and other organisms
☒	☐ Clinical data
☒	☐ Dual use research of concern
☒	☐ Plants

Methods

n/a	Involved in the study
☒	☐ ChIP-seq
☒	☐ Flow cytometry
☒	☐ MRI-based neuroimaging

Antibodies

Antibodies used

GAD-67 was from Merck Millipore (catalog no. MAB5406), Alexafluor 405-conjugated goat-anti-mouse antibody was from Life Techologies (cat. no. A31553)

Validation

The GAD-67 mouse antibody was from Merck Millipore (catalog no. MAB5406) and it was validated by the manufacturer. It is derived from Clone 1G10.2 ZooMAb mouse recombinant monoclonal antibody that specifically detects Glutamate decarboxylase 1 (GAD 67). Quality Control Testing Routinely evaluated by immunohistochemistry by SKNSH cell lysate. Immunohistochemistry(paraffin) Analysis: Representative staining pattern and morphology of GAD67 in somatosensory 1, barrel field of the mouse cerebral cortex. All brown spots are Lamina VI a neurons. No Epitope retrieval was necessary. This lot of the antibody was diluted to 1:500, using IHC Select® Detection with HRP-DAB. Optimal Staining pattern/morphology of GAD67: Mouse Brain

References
1. Ladewig, Julia, et al. (2008). Stem Cells.
2. King, A E, et al. (2006). J Comp Neurol. 498:277-94.
3. Ling, L. L., et al. (2005). Neuroscience.132:1103-1113.
4. Watanabe, K, et al. (2005). Nature Neurosci. 8:288-296.
5. Varea, E., et al. (2005). Neuroscience. (2005)136:435-443

Animals and other research organisms

Policy information about studies involving animals; ARRIVE guidelines recommended for reporting animal research, and Sex and Gender in Research

Laboratory animals

Rattus norvegicus of the Sprague Dawley strain (4-6 weeks old) and of the Wistar strain (14-19 days old), both were in house bred.

Wild animals

No wild animals were used in this study

Reporting on sex

Males and females, as specified in supplementary note 1

Field-collected samples

No field collected samples were used in the study

Ethics oversight

All animal handling procedures have been approved by the Veterinary Service of the Canton of Vaud (Authorizations VD2745, VD3205).

Note that full information on the approval of the study protocol must also be provided in the manuscript.
